# TraR, a Homolog of a RNAP Secondary Channel Interactor, Modulates Transcription

**DOI:** 10.1371/journal.pgen.1000345

**Published:** 2009-01-16

**Authors:** Matthew D. Blankschien, Katarzyna Potrykus, Elicia Grace, Abha Choudhary, Daniel Vinella, Michael Cashel, Christophe Herman

**Affiliations:** 1 Department of Molecular and Human Genetics, Baylor College of Medicine, Houston, Texas, United States of America; 2 Laboratory of Molecular Genetics, National Institute of Child Health and Human Development, National Institutes of Health, Bethesda, Maryland, United States of America; 3 Department of Molecular Virology and Microbiology, Baylor College of Medicine, Houston, Texas, United States of America; Stanford University, United States of America

## Abstract

Recent structural and biochemical studies have identified a novel control mechanism of gene expression mediated through the secondary channel of RNA Polymerase (RNAP) during transcription initiation. Specifically, the small nucleotide ppGpp, along with DksA, a RNAP secondary channel interacting factor, modifies the kinetics of transcription initiation, resulting in, among other events, down-regulation of ribosomal RNA synthesis and up-regulation of several amino acid biosynthetic and transport genes during nutritional stress. Until now, this mode of regulation of RNAP was primarily associated with ppGpp. Here, we identify TraR, a DksA homolog that mimics ppGpp/DksA effects on RNAP. First, expression of TraR compensates for *dksA* transcriptional repression and activation activities in vivo. Second, mutagenesis of a conserved amino acid of TraR known to be critical for DksA function abolishes its activity, implying both structural and functional similarity to DksA. Third, unlike DksA, TraR does not require ppGpp for repression of the *rrnB* P1 promoter in vivo and in vitro or activation of amino acid biosynthesis/transport genes in vivo. Implications for DksA/ppGpp mechanism and roles of TraR in horizontal gene transfer and virulence are discussed.

## Author Summary

Control of gene expression is central for cell operation. Transcription regulation is a first step to control gene expression and is largely mediated by DNA-binding factors. These recruit or prevent RNA polymerase binding to promoters of specific genes. Recently, a novel way to control transcription has emerged from studying nutritional stress in bacteria. In this case, a small nucleotide effector, ppGpp, with the help of a protein DksA, interacts with the secondary channel of RNAP, affecting RNA polymerase kinetics at promoters without binding to specific DNA sequences. This interaction results in up-regulation and down-regulation of genes involved in responding to nutritional stress. This work describes TraR, a factor found on conjugative plasmids that can regulate gene expression similarly to DksA, but in the absence of any nucleotide effector, like ppGpp. Thus, regulation of transcription similarly to DksA/ppGpp may be a more general mechanism. The presence of TraR on conjugative plasmids suggests a role for TraR in pathogenicity, virulence, and antibiotic resistance. These observations should provide a basis for new studies designed to combat antibiotic resistance and virulence in emerging pathogens.

## Introduction

The ability to respond to changes in nutritional environment is a universal need inherent in all cells and is characterized by rapid global changes in gene expression. Regulation of transcription initiation is a central way to control gene expression and is largely achieved through the use of DNA-binding proteins (activators and repressors) restricted to distinct promoters through recognition of specific DNA elements. Study of the nutritional response in *Escherichia coli* has detailed a novel mechanism of modulating transcription initiation, both positively and negatively, through the use of a single small nucleotide effector, guanosine tetraphosphate (ppGpp), that interacts with RNA polymerase [1]. In *E. coli*, the accumulation of ppGpp causes rapid effects on transcription; ppGpp binds to RNA polymerase, provoking an alteration in transcription kinetics that is proposed to result from a reduction in open complex stability [2]. Such effects include, but are not limited to, upregulation of amino acid biosynthesis and transport genes, as well as genes involved in stasis/stress survival, and downregulation of translational components such as rRNA and tRNA genes [3].

Recently, an additional factor, DksA, has been shown to potentiate the action of ppGpp on RNAP both in vitro and in vivo [4]–[6]. The loss of either ppGpp or DksA results in similar, though not identical, phenotypes including the downregulation of several amino acid biosynthetic pathways, and the inability to negatively regulate ribosomal RNA transcription [7],[8]. Separate from mediating the stringent response, DksA has roles in other processes including chromosome segregation, DNA repair, protein folding, bacterial motility, virulence, and the expression of type 1 fimbriae [7]–[14]. The crystal structure of DksA has been determined and shows that the 151 amino acid-long protein folds into three distinct structural domains: an N-terminal region containing two α-helices (coiled coil), a globular domain with a C4 Zn^+2^ finger motif, and a short C-terminal helix [15]. DksA is structurally analogous to GreA and GreB, transcriptional anti-pausing/fidelity factors that are homologs of the eukaryotic TFIIS [16]–[19]. The Gre factors bind RNAP and protrude their coiled coils deep into the secondary channel toward the active site [20],[21]. DksA also binds RNAP, and it has been suggested that, similar to the Gre factors, DksA could also interact with the secondary channel. This is supported by a growing body of evidence indicating that the DksA and Gre proteins compete in vivo for the same substrate, the secondary channel of RNAP [22],[23]. A proposed mechanism of action for DksA positions the coiled coil region deep within the RNAP secondary channel near the active site. At the tip of the coiled coil region, two invariant aspartic acid residues, Asp71 and Asp74, are thought to coordinate the ppGpp bound Mg^+2^ ion to effectively position ppGpp near the active site, and allow it to exert its transcriptional modulation effects [15]. Mutation of these two conserved aspartic acid residues abolishes DksA’s ability to modulate transcription with ppGpp [15]. The proposed DksA mechanism remains highly speculative, and it is unknown exactly how ppGpp and DksA influence each other or how their binding alters RNAP activity. Furthermore, detailed mutational analysis of the defined RNAP binding site for ppGpp has cast doubt on the biological relevance of the placement of ppGpp in the original ppGpp/RNAP co-crystal structure [24].

Comparisons of DksA with sequence databases have previously found similarities of DksA to several bacteriophage ORFs and TraR, a protein found on conjugative plasmids that promote horizontal gene transfer (for alignments, refer to [15],[25]). Horizontal transfer of DNA allows the acquisition of new traits in the recipient bacterium, such as virulence or resistance to antibacterial agents [26]. The well-studied F plasmid of *E. coli* is considered a model for bacterial conjugation and is facilitated by a large protein complex, the F pilus, which bridges the donor and recipient cell membranes, enabling F plasmid DNA transfer [27]. The components of the F pilus, along with regulatory, accessory, and unknown factors, are encoded on the single 33-kb transfer (*tra*) operon [27]. *traR* encodes a gene in the downstream region of the *tra* operon that is dispensable for F plasmid transfer, at least under normal laboratory conditions [25],[28],[29]. The sequence homology of TraR to DksA, while weak (30% identity), raises the possibility that episomal TraR possesses some functional similarities to DksA.

In this study, we show that TraR modulates gene expression similarly to ppGpp/DksA, but in the absence of any nucleotide effector, like ppGpp. Expression of TraR compensates for *dksA* transcriptional repression and activation activities in vivo. Mutagenesis of a TraR amino acid corresponding to a critical residue for DksA function abolishes activity, implying structural similarity to DksA. Compensation by TraR is inhibited by overexpression of GreB, a factor known to interact with the RNAP secondary channel [21], suggesting, like ppGpp/DksA, that TraR also interacts similarly with RNAP. Surprisingly, unlike DksA, TraR does not require ppGpp for repression of the *rrnB* P1 promoter either in vivo or in vitro, or for activation of amino acid biosynthesis/transport promoters in vivo at physiological levels. The activity of TraR in the absence of ppGpp could provide clues on the mechanistic role of DksA in modulating RNA polymerase for at least several cellular processes. The implications of our findings on current models of DksA/ppGpp action will be discussed, as well as the implications for roles of episomal *traR* in conjugation, pathogenicity, and the evolution of gene expression.

## Results

### Endogenously Expressed TraR Functions in Amino Acid Biosynthesis

Since TraR shares limited sequence homology to the transcriptional modulator DksA, we hypothesized that the two proteins could possess some functional similarities. Loss of DksA function causes permanent downregulation of several amino acid biosynthetic and transport pathways and results in an inability of *E. coli* cells to grow on minimal media without supplementation of required amino acids [4],[6],[11]. If TraR shares functions with DksA, expression of TraR in a Δ*dksA* strain should compensate for the multiple auxotrophies. Indeed, when a mini-F plasmid (pOX38, see Supplemental Materials), which naturally contains *traR* in the transfer operon [28], was conjugated into a *dksA* null strain, prototrophic growth on minimal media was observed (Table 1). The restoration to prototrophy by the F plasmid is TraR-dependent since complete deletion of *traR* abolishes the prototrophy observed in the Δ*dksA* F factor strain. The ability of TraR to compensate for the multiple auxotrophies of Δ*dksA* suggests that TraR possesses functional similarities to DksA. Furthermore, overexpression of GreB (from an inducible pTrc plasmid, see Supplemental Materials), a factor known to interact with the RNAP secondary channel [21], reduced the prototrophic compensation by TraR expressed from the F episome (Table 1). GreB was previously shown to compete with DksA and does not rescue Δ*dksA* auxotrophies [22],[23] (unpublished data). The competition of GreB with TraR suggests that TraR interacts with the secondary channel.

**Table 1:** Expression of TraR Rescues Δ*dksA* Amino Acid Auxotrophies.

Genotype	% cfu M9-glu / M9-glu-CAA	% cfu M9-glu / M9-glu-CAA
	(no IPTG)	(0.1 mM IPTG)*
Δ*dksA*	1.4×10^−3^±1.2×10^−4^	NA
Δ*dksA* [F]	98±9.8	NA
Δ*dksA* [F Δ*traR*]	3.4×10^−4^±2.9×10^−4^	NA
Δ*dksA* [F] [pGreB]	100±9	6.4±1.1
Δ*dksA* [pControl]	1.8×10^−4^±1.3×10^−4^	2.3×10^−4^±6.1×10^−5^
Δ*dksA* [pDksA]	103±7.2	101±6.2
Δ*dksA* [pTraR]	105±3.2	103±6.5
Δ*dksA* [pTraR-D4N]	1.1×10^−3^±4.4×10^−4^	14±9.6

Plating efficiencies of colony forming units (cfu) on M9-glucose (glu) vs. M9-glucose (glu)-casamino acids (CAA), ±IPTG. Percentages depict means±standard deviation of the mean from 3 independent determinations. TraR, expressed either endogenously from the F plasmid or ectopically from multicopy plasmids (a pTrc99A derivative), rescues Δ*dksA* amino acid auxotrophies. Overexpression of GreB, a factor known to interact with the RNAP secondary channel [21], inhibited the compensation by TraR. (^*^) 1 mM IPTG was used for the GreB experiment.

### Predicted Structural Features of TraR

**Figure 1:**
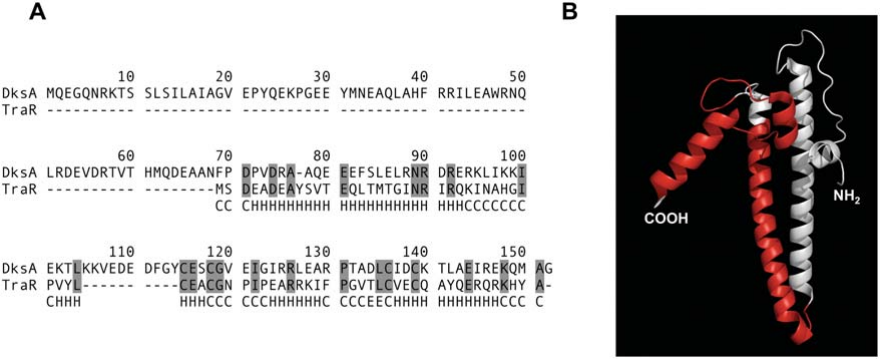
Sequence Alignment Between DksA and TraR. (A) Alignment of TraR sequence with DksA. TraR secondary structure prediction was performed with the PSIPRED prediction method [56] and is labeled below the TraR sequence. H = helix, E = strand, and C = loose coil. PSIPRED predicts a high probability of an initial helix present in TraR. (B) Model of DksA [15] highlighting (red shading) residues aligned between TraR and DksA.

Given the sequence homology between TraR and DksA (for alignments see Figure 1A, or refer to [15],[25]) and our demonstration that TraR rescues defects associated with loss of DksA, it is likely that TraR and DksA share structural similarities. Sequence analysis predicts that TraR possesses a globular domain with the C4 Zn^+2^ finger motif, characteristic of the DksA family [15]. The sequence of TraR also begins with the two conserved aspartic acid residues that are important for DksA function [15]. Unlike DksA however, TraR is 73 amino acids long, making TraR approximately half the size of DksA. Secondary structure prediction suggests that TraR starts with a long helical structure, which could correspond to half of the coiled coil domain of DksA and the length of this predicted helix would be shorter than the corresponding region of DksA (Figure 1A,B).

### Ectopically Expressed TraR Upregulates Amino Acid Biosynthesis

To address whether TraR is sufficient to compensate for *dksA* defects, *traR* and *dksA* were separately cloned onto a multi-copy plasmid under an inducible pTrc promoter (pBA169, see Supplemental Materials). Ectopic expression of TraR rescued the inability of Δ*dksA* cells to grow in the absence of required amino acids. The plating efficiencies of Δ*dksA* cells containing TraR or DksA plasmids grown on M9-glucose plates compared to those grown on M9-glucose-casamino acid plates approached 100%, approximately 5 orders of magnitude higher than with the control plasmid (Table 1). Interestingly, both uninduced and induced pTraR and pDksA plasmids provided a complete rescue of the Δ*dksA* auxotrophy, suggesting that only a few copies of TraR or DksA in the cell are needed to restore appropriate regulation of amino acid biosynthesis and transport. To address the functional similarities between TraR and DksA, the second aspartic acid residue of TraR, D6, which corresponds to the invariant Asp74 of DksA (see above), was mutated to asparagine. As shown in Table 1, TraR(D6N) was no longer able to fully compensate for Δ*dksA* auxotrophy. This observation emphasizes the functional importance of this aspartic acid residue conserved in TraR and DksA.

### TraR Activates the Stringently Induced *livJ* Promoter

**Figure 2:**
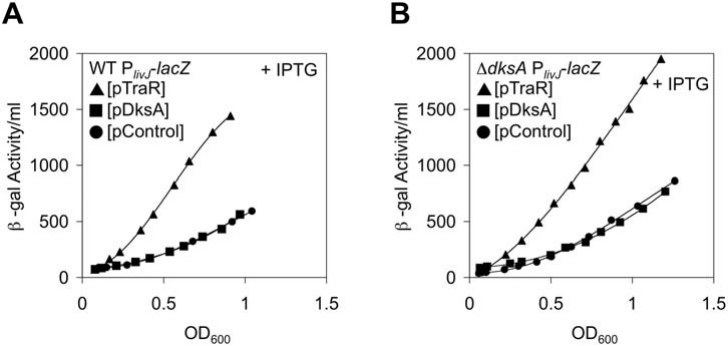
Activation of the *livJ* Promoter by Expression of TraR. β-galactosidase activity of the P*_livJ_*-*lacZ* promoter fusion in LB media. Differential activity of β-galactosidase plotted. (A) Wild-type background. Induced (0.1 mM IPTG) expression of the TraR (triangles) activates the *livJ* promoter. WT DksA plasmid (squares) and control plasmid (circles). Rates of β-galactosidase synthesis in exponential phase (from OD_600_ 0.2 to 0.6) are ∼520, 480, and 1010 β-gal activity/OD_600_ for pControl, pDksA, and pTraR, respectively. R^2^ values>0.95 in this linear range. (B) As in A, but a Δ*dksA* background. Rates of β-galactosidase synthesis in exponential phase (from OD_600_ 0.2 to 0.6) are ∼520, 670, and 1500 β-gal activity/OD_600_ for pControl, pDksA, and pTraR, respectively. R^2^ values>0.95 in this linear range.

To examine in more detail the restoration of prototrophy by TraR in a Δ*dksA* strain, activation of the *livJ* promoter was explored. *livJ* encodes a transporter for branched-chain amino acids and is activated by ppGpp/DksA [6]. β-galactosidase assays with a wild-type P*_livJ_*-*lacZ* fusion strain yielded strong activation of the *livJ* promoter by induction of TraR in exponential growth (Figure 2A). In contrast, we observed little effect with DksA overexpression. The lack of seeing effects with DksA, which is ppGpp-dependent with respect to the *livJ* promoter [6], is probably due to the low levels of ppGpp present in rich media during exponential growth. Identical results were obtained when the experiments were repeated in a Δ*dksA* background, supporting that TraR can work independently of DksA in the activation of *livJ* (Figure 2B). Thus, TraR, unlike DksA, is able to activate the *livJ* promoter in exponential phase.

### TraR Inhibits Ribosomal RNA Transcription

**Figure 3:**
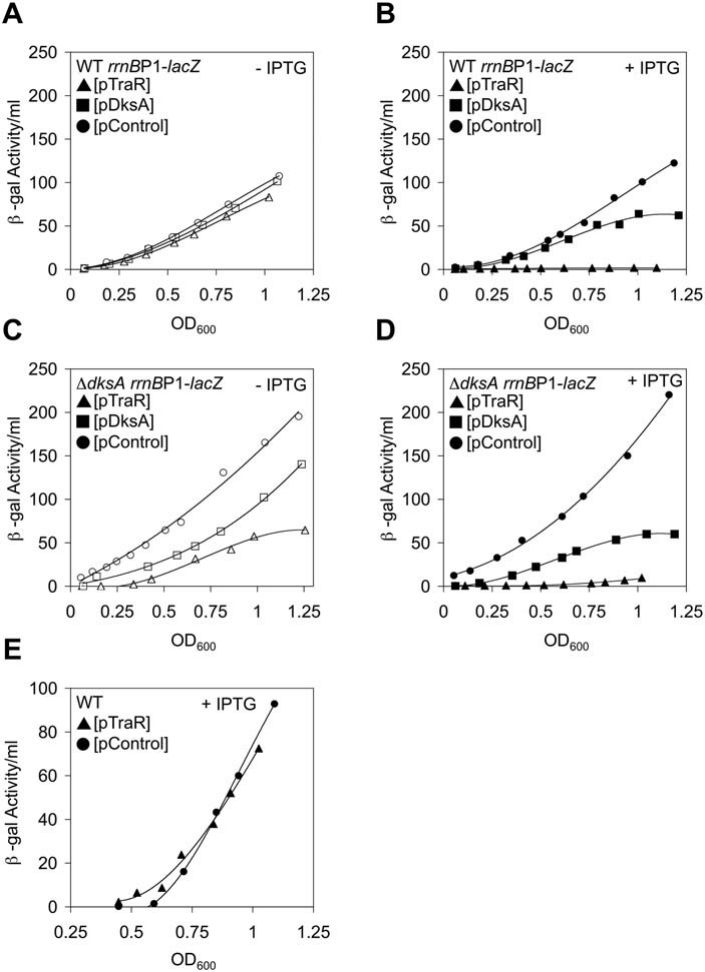
Inhibition of the *rrnB* P1 Promoter by Ectopically Expressed TraR. Differential β-galactosidase activity of the *rrnB* P1-*lacZ* promoter fusion in LB media. (A,B) Wild-type background. (A) Effects of uninduced ectopic expression of TraR (open triangles) or DksA (open squares) on *rrnB* P1 activity. Plasmid control indicated by open circles. Rates of β-galactosidase synthesis in exponential phase (from OD_600_ 0.2 to 0.6) are ∼100, 100, and 84 β-gal activity/OD_600_ for pControl, pDksA, and pTraR, respectively. R^2^ values>0.95 in this linear range. (B) Induced (0.1 mM IPTG) ectopic expression of TraR (triangles) or DksA (squares) represses *rrnB* P1 activity. Circles, plasmid control. Rates of β-galactosidase synthesis in exponential phase (from OD_600_ 0.2 to 0.6) are ∼95, 67, and 2.0 β-gal activity/OD_600_ for pControl, pDksA, and pTraR, respectively. R^2^ values>0.95 in this linear range. (C,D) Δ*dksA* background. (C) Repression of β-galactosidase activity of *rrnB* P1-*lacZ* from uninduced ectopic expression of TraR (open triangles) or DksA (open squares) in the Δ*dksA* cells. Plasmid control indicated by open circles. Rates of β-galactosidase synthesis in exponential phase (from OD_600_ 0.2 to 0.6) are ∼140, 84, and 88 β-gal activity/OD_600_ for pControl, pDksA, and pTraR, respectively. R^2^ values>0.95 in this linear range. (D) Induced (0.1 mM IPTG) ectopic expression of TraR (triangles) or DksA (squares) represses *rrnB* P1 activity in Δ*dksA*. Circles, plasmid control. Rates of β-galactosidase synthesis in exponential phase (from OD_600_ 0.2 to 0.6) are ∼140, 80, and 3.3 β-gal activity/OD_600_ for pControl, pDksA, and pTraR, respectively. R^2^ values>0.95 in this linear range. (E) TraR does not affect the P*_lac_* promoter. β-galactosidase activity assays of the wild-type *lac* operon. TraR (triangles) induced (0.1 mM IPTG) from a multi-copy plasmid. Control plasmid indicated by circles.

DksA is a pleiotropic regulator of transcription with positive and negative effects on a wide array of genes [4],[6],[7],[13],[15]. One of the more extensively characterized effects of DksA (and ppGpp) is the negative regulation of ribosomal RNA (rRNA) accumulation [22],[23]. Since TraR was shown to compensate for DksA in the positive regulation of amino acid genes, we explored whether TraR can also negatively affect the transcription of rRNA. Since expression of both TraR and DksA from the uninduced plasmids fully compensated for Δ*dksA* auxotrophies (above), uninduced levels of TraR and DksA on the *rrnB* P1 promoter were examined first. Uninduced levels of TraR expressed from the plasmid did not cause a significant negative effect on *rrnB* P1-*lacZ* activity compared to the control plasmid in a wild-type background (Figure 3A). We next examined *rrnB* P1-*lacZ* activity during IPTG-induced TraR and DksA overexpression. TraR reduced *rrnB* P1 transcription to negligible levels immediately after induction in early exponential growth (Figure 3B). This inhibition of transcription by overexpression of TraR was specific to the *rrnB* P1 promoter since no significant effect was observed on the wild-type *lac* promoter (Figure 3E). DksA, when overexpressed, also had inhibitory effects on the *rrnB* P1-*lacZ* construct compared to the control plasmid, though the effect was much less pronounced than that of TraR (Figure 3B). The DksA-mediated repression became greater as cells exited exponential growth, corresponding to the accumulation of ppGpp as cells approach stationary phase [1],[30]. The repression of rRNA transcription exerted by TraR on *rrnB* P1 activity in a wild-type strain strongly reinforces the suggestion that TraR can act like DksA.

Since TraR expressed from the mini-F compensated for the Δ*dksA* amino acid auxotrophies in minimal media, we asked whether endogenous expression of *traR* from the mini-F plasmid could also affect the *rrnB* P1 promoter under the same conditions. Upon mating *traR* ^+^ and Δ*traR* mini-F plasmids into a wild-type *rrnB* P1-*lacZ* fusion strain, we observed that the presence of TraR negatively affected activity from of *rrnB* P1 (Supplemental Materials, Figure S1). Although this effect was modest, a large effect of endogenously expressed *traR* on the *rrnB* P1 promoter was not expected since the presence of the F episome does not significantly diminish growth rate and the *tra* operon is only partially derepressed on the F plasmid in *E. coli* [31],[32]. That endogenous *traR* expressed from the F plasmid rescues the Δ*dksA* amino acid auxotrophies near 100%, but the F plasmid’s effects on the *rrnB* P1 promoter are modest, suggests that activation of amino acid synthesis is more sensitive to a lower concentration of TraR than the inhibition of rRNA synthesis. While activation of several amino acid genes could conceivably take only a few copies of TraR, inhibition of even small portion of rRNA transcription, which constitutes the majority of all transcription in *E. coli* [33],[34] would take many more copies of TraR.

### The Absence of DksA Enhances TraR Inhibition of rRNA Transcription

Given that derepression of rRNA synthesis is observed in *dksA* mutants [5],[22] and that TraR compensates for the amino acid requirements of a Δ*dksA* strain, we next asked whether the inhibitory effect of TraR on *rrnB* P1 is independent of DksA and whether TraR can fully compensate for the derepression seen in a Δ*dksA* strain. As expected, *rrnB* P1-*lacZ* activity was derepressed about 50% in Δ*dksA* compared to its *dksA* ^+^ counterpart during exponential growth (compare pControl curves from Figure 3A and Figure 3C). Uninduced pDksA complemented the Δ*dksA rrnB* P1 derepression to wild-type levels. Furthermore, uninduced pTraR not only fully compensated for Δ*dksA*, but also again exerted stronger inhibition of *rrnB* P1 transcription compared to DksA (Figure 3C). We then studied the effects of overexpression of TraR and DksA on *rrnB* P1-*lacZ* activity in a Δ*dksA* background. As in the uninduced experiments, *rrnB* P1 was derepressed in Δ*dksA* cells, and overexpression of DksA fully complemented the derepression to wild-type levels during exponential growth (Figure 3D). Overexpression of TraR in the Δ*dksA rrnB* P1-*lacZ* background again resulted in a strong repression of *rrnB* P1-*lacZ* activity immediately after induction during early exponential growth (Figure 3D). As seen for the uninduced plasmids, the magnitude of TraR-mediated repression in early exponential growth was greater in the Δ*dksA* background (40-fold) than wild-type (27-fold) (Figure 3D and Figure 3B, respectively). This difference had a variation of about 10% and matches the 50% *rrnB* P1 derepression observed in Δ*dksA.* The enhanced effects of TraR in the Δ*dksA* strain further reveal shared functions between TraR and DksA. The combined *rrnB* P1 results not only extend the functional similarity of TraR and DksA to negative regulatory effects, but also further demonstrate that TraR may function as a more effective modulator of transcription than DksA in exponential phase.

### TraR Inhibits Growth

**Figure 4:**
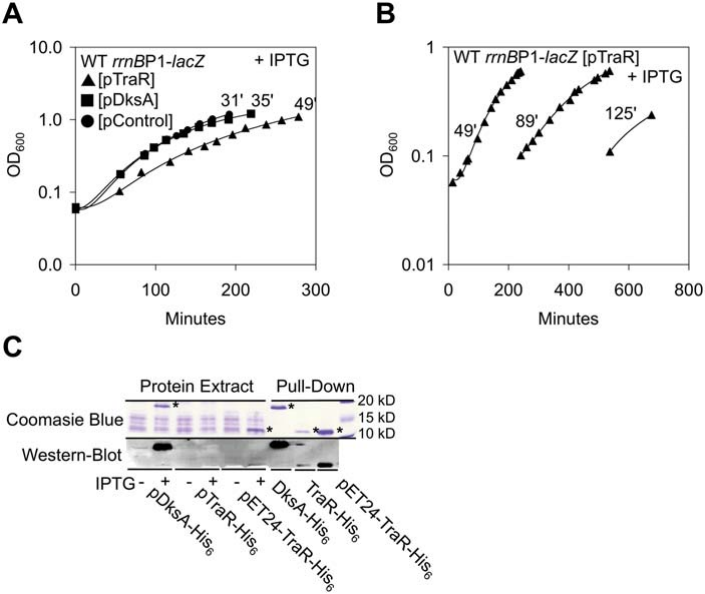
TraR Inhibits Growth and Is Expressed Less than DksA. (A,B) TraR Inhibits Growth. (A) Semilog plot of OD_600_ vs. growth time resulting from induced (0.1 mM IPTG) expression of TraR (triangles) or DksA (squares) in the WT *rrnB* P1-*lacZ* background grown in LB-ampicillin at 32°C. Circles, plasmid control. (B) Figure depicting increasing doubling times (early logarithmic growth) resulting from successive dilutions in LB containing IPTG of a logarithmic culture induced for TraR (triangle). pControl and pDksA do not inhibit growth when treated similarly (35, 33, 35 minutes and 36, 36, 37 minutes were the respective doubling times, see Figure S2, Supplemental Materials). (C) Steady state levels of TraR expressed from pTraR-His_6_ are lower than corresponding DksA levels. (Left) Protein extracts from indicated plasmids (±IPTG) were separated by SDS-PAGE (12%) and detected by Coomassie staining (upper left) or Western blotting using an anti-His_6_ antibody (lower left). Bands corresponding to TraR and DksA are indicated by asterisks. (Right) TraR-His_6_ and DksA-His_6_ were pulled-down from identical amounts of protein extracts made after two hours of induction, subjected to SDS-PAGE, and detected by Coomassie blue staining (upper right) or Western blotting (anti-His_6_ antibody) (lower right). Purification plasmid pET24a-TraR-His_6_ was used as a positive control to visualize TraR-His_6_ in both assays.

The strong downregulation of transcription from the *rrnB* P1 promoter by TraR is expected to decrease the growth rate due to an inhibition of ribosome synthesis [1],[35]. Hence, we asked whether TraR could inhibit bacterial growth. Figure 4A shows growth curves for WT *rrnB* P1-*lacZ* strains overexpressing TraR, DksA, or control plasmids. When overexpressed, TraR is shown to slow growth compared to DksA and control plasmids. Specifically, the doubling time increased after the second generation from 31 minutes (control plasmid) to 49 minutes when TraR is expressed. Little growth difference resulted from DksA overexpression in logarithmic growing cells (doubling time 35 minutes), highlighting an important difference between the two homologs.

With near complete inhibition of ribosome synthesis and cell reliance on pre-existing ribosomes, it was further predicted that doubling time would be progressively reduced due to dilution of ribosome pools during subsequent growth. To test this, we diluted an overnight culture (pTraR) one hundred-fold in LB media containing IPTG and measured growth over time. When the logarithmic culture reached an OD_600_ of 0.6, a six-fold dilution was performed in the same media and growth continued to be monitored. A second six-fold dilution was performed when the culture reached OD_600_ 0.6 a third time. The doubling time increased from 49 to 89 and 125 minutes, respectively, and showed an overall 4-fold increase after being carried out for seven doublings (Figure 4B). In agreement with the growth results above (Figure 4A), neither the pDksA (doubling times of 36, 36, and 37 minutes) or control plasmid (doubling times of 35, 33, and 35 minutes) resulted in any significant growth defects when serially diluted (see Supplemental Materials, Figure S2).

Despite lowered growth rates, cells never stopped growing and appeared to undergo an adaptation to the strong *rrnB* P1 repression by TraR. Assaying for *rrnB* P1 activity in WT *rrnB* P1-*lacZ* cells overexpressing TraR after overnight growth, we observed that *rrnB* P1 activity was still repressed, although not as strongly as before (data not shown). This new low level of *rrnB* P1 transcription may result from a feedback inhibition mechanism. Complete inhibition of the protein synthesis machinery by TraR would ultimately inhibit its own production, resulting in upregulation of *rrnB* P1 transcription. Protein synthesis would resume until TraR reaccumulates. This negative feedback loop would produce slower balanced growth. Such a scenario is also likely for the colonies forming on plates containing IPTG. The data described above indicate that TraR, unlike DksA, has pronounced negative effects on cells during logarithmic growth. These negative effects on bacterial growth became more pronounced as the cells continued to divide and were likely caused by dilution of the pre-existing ribosomes after each cell division.

### TraR Is a Potent Regulator of rRNA Transcription in Exponential Growth

Western blots were performed to measure induced levels of TraR and DksA in order to ascertain whether differences in activation and repression activity were due to differences in protein expression. We first constructed C-terminal epitope-tagged TraR-His_6_ and DksA-His_6_ fusions and confirmed their wild-type behavior in vivo (data not shown). Western blot analysis, as well as Coomassie blue staining, showed the presence of a DksA band upon induction (∼8-fold higher than wild-type DksA levels, data not shown), but no band corresponding to TraR was detected (Figure 4C). To rule out complications inherent to the Western blot, we performed pull-down experiments to concentrate and measure the relative amount of TraR-His_6_ and DksA-His_6_ present in extracts made after two hours of induction. As shown in Figure 4C, the amount of TraR pulled-down is significantly less than DksA. As a positive control for Western blotting and pull-downs, TraR induced from our purification plasmid (pET24a-TraR-His_6_, see below) was successfully detected in both assays. Overall, these data suggest that low levels of TraR, compared to DksA, can achieve both strong activation of amino acid biosynthesis and potent inhibition of rRNA accumulation in exponential growth.

### TraR Functions Independently of ppGpp In Vivo

**Figure 5:**
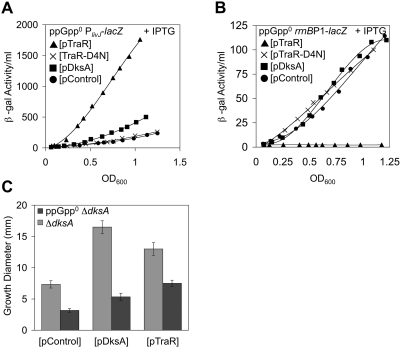
TraR Functions In Vivo Without ppGpp. (A) Strong activation of β-galactosidase activity from P*_livJ_*-*lacZ* in LB media with induced (0.1 mM IPTG) expression of TraR (triangles) in Δ*relA* Δ*spoT* (ppGpp^0^) cells. DksA and control plasmids indicated by squares and circles, respectively. The ppGpp-independent activation is dependent on the 6^th^ Asp residue of TraR mutated in pTraR-D6N (X). Rates of β-galactosidase synthesis in exponential phase (from OD_600_ 0.2 to 0.6) are ∼140, 400, 1900, and 150 β-gal activity/OD_600_ for pControl, pDksA, pTraR, and pTraR-D6N, respectively. R^2^ values>0.95 in this linear range. (B) Inhibition of β-galactosidase activity from *rrnB* P1-*lacZ* in LB media with induced (0.1 mM IPTG) expression of TraR (triangles) in a Δ*relA* Δ*spoT* (ppGpp^0^) background. DksA and control plasmids indicated by squares and circles, respectively. Repression by TraR is abolished by mutation of 6^th^ Asp residue (pTraR-D6N, diamonds). Rates of β-galactosidase synthesis in exponential phase (from OD_600_ 0.2 to 0.6) are ∼82, 120, 0.0, and 110 β-gal activity/OD_600_ for pControl, pDksA, pTraR, and pTraR-D6N respectively. R^2^ values>0.95 in this linear range. (C) Ectopic uninduced expression of DksA or TraR suppress the cell motility defects of either Δ*dksA* or Δ*relA* Δ*spoT* (ppGpp^0^) Δ*dksA* cells. Strains were inoculated on low agar plates (0.375%) and grown for ∼24 hours at room temperature, at which the diameters of resulting growth areas produced by motile cells were measured (see Figure S3 in Supplemental Materials for representative picture). Means±standard deviation plotted.

It is well established that DksA influences the regulatory activities of ppGpp. Cells lacking DksA share a wide variety of phenotypes with those deficient for ppGpp [7]. Because of the intimacy between DksA and ppGpp, we sought to examine whether this characteristic extends to TraR. Our first hints that TraR and DksA might differ with respect to ppGpp were the observed potent *livJ* activation and *rrnB* P1 repression caused by TraR in early exponential growth, when little ppGpp is present [1],[30]. To directly examine the effects of ppGpp on TraR function, we examined *livJ* promoter activity in a Δ*relA* Δ*spoT* (ppGpp^0^) strain, which lacks both synthetases for ppGpp production [36]. As observed previously [7], ppGpp deficiency caused decreased expression of the *livJ* promoter compared to wild-type (compare plasmid controls in Figure 5A and 2A), and DksA overexpression compensated for this defect (Figure 5A). It is interesting and unknown why the *livJ* promoter has lower activity in a ppGpp^0^ background. It is also unknown how overexpression of DksA restores the *livJ* activity to wild-type levels in this background. Induced TraR expression in ppGpp^0^ cells resulted in strong activation of the *livJ* promoter identical to a ppGpp^+^ background (Figure 5A and 2A,B, respectively).

Previous studies have shown the functional importance of the conserved, invariant aspartic acid residues (D71 and D74, tip of coiled coil domain) of DksA. To address the functional importance of these residues with respect to the TraR, the second aspartic acid residue of TraR was changed to asparagine (D6N) and assayed for P*_livJ_*-*lacZ* activity in the absence of ppGpp^0^ (Figure 5A). Altering this second aspartic acid residue abolished the strong activation seen with TraR expression, supporting the shared functional importance of the invariant aspartic acid residues in both TraR and DksA.

We next examined the effects of ppGpp on the negative regulatory aspects of TraR by measuring TraR-mediated inhibition of *rrnB* P1 activity in the Δ*relA* Δ*spoT* (ppGpp^0^) background. As expected, loss of ppGpp disrupted *rrnB* P1 repression mediated by DksA, resulting in activity identical to the control plasmid (Figure 5B). However, induction of TraR in the ppGpp^0^ strain caused an immediate and striking downregulation of the *rrnB* P1 promoter as observed in a ppGpp^+^ strain (Figure 5B and Figure 3B, respectively). Therefore, with respect to the *rrnB* P1 promoter, TraR and DksA are not entirely functionally interchangeable because TraR appears to be ppGpp-independent while DksA is ppGpp-dependent. Interestingly, the D6N mutation of TraR, which alters a conserved aspartic acid residue, completely abolished the *rrnB* P1 repression in the ppGpp^0^ strain (Figure 5B), again emphasizing the importance of the invariant aspartic acid residues for TraR function.

We next asked whether TraR could also satisfy the multiple amino acid requirements of a ppGpp^0^ strain [36]. Table 2 shows the TraR-mediated rescue of Δ*relA* Δ*spoT* (ppGpp^0^) auxotrophies. The control plasmid in the ppGpp^0^ strain exhibited the expected very low plating efficiency (10^−3^%) on M9-glucose vs. M9-glucose-casamino acid plates. Also, no compensation of the ppGpp^0^ auxotrophies was observed when TraR was not overexpressed. In contrast, IPTG-induced levels of TraR resulted in an 85% plating efficiency on M9-glucose plates in the ppGpp^0^ strain. DksA overexpression in the ppGpp^0^ strain exhibited only a weak rescue for growth on M9-glucose. The resulting plating efficiency of 0.5% was similar to results reported by Magnusson et al. [7] in a MG1655 ppGpp^0^ background. However, their observation was noted to be strain specific (see Discussion).

**Table 2:** TraR Rescues ppGpp^0^ Amino Acid Auxotrophies.

Genotype	% cfu M9-glu / M9-glu-CAA	% cfu M9-glu / M9-glu-CAA
	(no IPTG)	(0.1 mM IPTG)
ppGpp^0^ [pControl]	3.7×10^−3^±5.4×10^−3^	3.8×10^−3^±5.3×10^−3^
ppGpp^0^ [pDksA]	1.4×10^−3^±8.8×10^−4^	0.50±0.30
ppGpp^0^ [pTraR]	3.6×10^−3^±8.6×10^−4^	85±7.7
ppGpp^0^ Δ*dksA* [pControl]	7.5×10^−3^±4.6×10^−3^	5.6×10^−3^±3.3×10^−3^
ppGpp^0^ Δ*dksA* [pDksA]	1.6×10^−3^±1.5×10^−3^	0.24±0.046
ppGpp^0^ Δ*dksA* [pTraR]	47±3.5	99±0.5
ppGpp^0^ Δ*dksA* [F]	1.3×10^−5^±1.2×10^−5^	NA
ppGpp^0^ Δ*dksA* [F Δ*traR*]	2.4×10^−5^±1.7×10^−5^	NA

Plating efficiencies (M9-glucose vs. M9-glucose-casamino acids, ±IPTG) for either ppGpp^0^ *dksA* ^+^ cells or ppGpp^0^ Δ*dksA* cells with uninduced and induced expression of the plasmid control, DksA, or TraR or with the presence of a F plasmid (*traR* ^+^ or Δ*traR*). Means of 3 independent determinations are plotted±standard deviation.

The predicted structural homology and functional similarities between TraR and DksA suggest that TraR interacts with the RNAP secondary channel, further supported by the in vivo GreB competition data above (Table 1). It is likely that TraR, because of low expression levels, has to compete for the secondary channel with endogenous DksA to exert its positive biosynthetic effects. If this is correct, then deleting *dksA* might not only enhance the ability of TraR to rescue the multiple ppGpp^0^ amino acid auxotrophies, but also provide evidence of similar binding to RNAP. As predicted, the uninduced TraR plating efficiency on M9-glucose increased from 10^−3^% in the ppGpp^0^ *dksA* ^+^ background to 47% in the ppGpp^0^ Δ*dksA* background (Table 2). For the DksA and control plasmids, the loss of *dksA* to the ppGpp^0^ caused no noticeable effects compared to the ppGpp^0^ *dksA* ^+^ strain. This striking difference in compensation activity suggests competitive binding between TraR and DksA to RNA polymerase, supporting a modulatory role of TraR within the RNA polymerase secondary channel. In addition, these data further support the ppGpp-independent nature of gene regulation by TraR.

No suppression of ppGpp^0^ Δ*dksA* amino acid auxotrophies was observed with the F plasmid (Table 2), contrasting the full suppression of Δ*dksA* amino acid auxotrophies by endogenous *traR*. The reason for this discrepancy remains unknown. Considering that low uninduced levels of cloned *traR* can rescue ppGpp^0^ Δ*dksA* amino acid auxotrophies, one possibility is TraR expression from the F plasmid is reduced in a ppGpp^0^ background.

It has been previously shown that bacterial motility is impaired in ppGpp^0^ cells, and that expression of DksA could suppress this motility defect [7]. DksA may activate motility by enhancing the competitiveness of the sigma factor, σ^F^, required for the production of flagella and chemotaxis [7],[37]. Uninduced pTraR, like DksA, activated motility in both Δ*dksA* and ppGpp^0^ Δ*dksA* backgrounds (Figure 5C).

The above results demonstrate that TraR in the absence of ppGpp activates *livJ* and rescues the multiple ppGpp^0^ amino acid auxotrophies. TraR also suppresses motility defects of ppGpp^0^ and Δ*dksA* cells. Furthermore, TraR alone causes rapid, near complete repression of rRNA transcription. These observations clearly identify important (and unexpected) differences between TraR and DksA.

### TraR Acts Independently of ppGpp In Vitro

**Figure 6:**
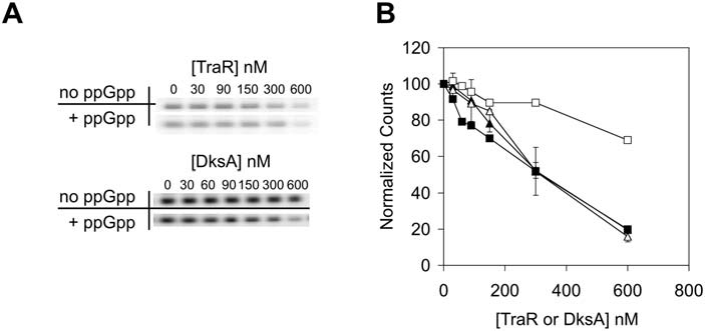
TraR Inhibits *rrnB* P1 Transcription In Vitro Independent of ppGpp. Single round transcriptions were performed in the presence (filled symbols) or absence (open symbols) of 250 µM ppGpp with increasing concentrations (0–600 nM) of TraR-His_6_ (triangles) or DksA-His_6_ (squares). Amounts of RNA were measured by phosphorimaging (A) and are quantified in (B), normalized to the equivalent units observed at TraR = 0 or DksA = 0 for each set of reactions separately. Means of 3 independent determinations of TraR (±standard deviation) and a DksA control experiment performed in parallel are plotted. In vitro DksA results agree with previously published data [5],[22].

The above in vivo results clearly demonstrate that TraR can both repress rRNA transcription and activate amino acid biosynthesis independently of ppGpp. To determine whether the ppGpp-independent action of TraR on the *rrnB* P1 promoter is direct, we performed a single round in vitro transcription assay with purified TraR (His_6_-tagged) or DksA (His_6_-tagged) as described in [22]. Addition of increasing amounts of TraR to the transcription mixture showed a linear inhibition of *rrnB* P1 transcription with or without ppGpp (Figure 6A,B). Using identical conditions for this range of concentrations, DksA only showed inhibition of *rrnB* P1 promoter in the presence of ppGpp (Figure 6A,B and [22] [5]). The in vitro data confirm that TraR alone can directly inhibit the *rrnB* P1 promoter in a ppGpp-independent manner.

## Discussion

Transcriptional control via interactions with the RNA polymerase secondary channel is an emerging field for both prokaryotes and eukaryotes [38]. Unlike classical regulators of transcription that bind to specific DNA sites associated with their target promoters, the transcription factor described in this work, similar to its homolog DksA, most likely employs the secondary channel of RNA polymerase to modulate gene expression. Here we show that a small protein, like TraR, can repress and activate gene expression similarly to DksA, but without any nucleotide effector, such as ppGpp. TraR can act similarly to DksA in both activation of amino acid biosynthetic and transport pathways and repression of rRNA transcription, and that overexpression of a known secondary channel interactor inhibits TraR’s compensation ability. However, TraR differs functionally from DksA in several important aspects. The effects of TraR on *rrnB* P1 activity occur much earlier in the growth cycle of *E. coli* and are noticeably stronger than those of DksA. The pronounced effects of TraR are not due to higher levels of TraR present in the cell, but result from an increased potency of TraR on transcription; very low levels of TraR exert stronger effect(s) in exponential growth compared to higher levels of DksA. The most surprising difference between TraR and DksA concerns ppGpp: DksA function is ppGpp-dependent at physiological levels while TraR acts independently of the nucleotide effector, in vitro, and this independence extends to both the positive and negative effects of TraR in vivo.

### TraR, a Putative Member of the RNAP Secondary Channel Interacting Proteins

TraR highlights a growing class of transcriptional regulators that may interact directly with the secondary channel. This class spans both prokaryotes and eukaryotes and includes DksA, GreA, GreB, TFIIS, Microcin J25, Gfh1, and Rnk [11], [15], [19]–[21], [39]–[43]. Other putative members are bacteriophage ORFs [15] and the uncharacterized *E. coli* ORF *ybiI*, which clusters in an iron metabolism operon. YbiI was identified through a BLAST search for sequences sharing homology to TraR and is capable of rescuing the amino acid auxotrophies of Δ*dksA*, but not of Δ*relA* Δ*spoT*, when expressed at high levels (data not shown). However, YbiI is more likely involved in iron metabolism. Secondary channel interactors can be sequence diverse, but structurally similar, as is the case between the Gre proteins and DksA [15],[20],[21]. Despite likely structural similarities throughout this class of regulators and their interactions inside the secondary channel, their transcriptional effects can vary with respect to initiation and their relationships to ppGpp. TraR, like DksA, is unique compared to the rest of the known secondary channel interacting proteins in that it functions in both positive and negative regulation of transcription initiation. GreB, for instance, has been shown to function similarly to DksA with respect to rRNA inhibition, but is unable to activate amino acid promoters [23]. GreA, on the other hand, activates rRNA transcription in vitro at the level of open complex formation, but not by altering the half-life of formed complexes [22]. The molecular basis for the differences in transcriptional initiation by these factors remains to be determined. Identification of factors like TraR will provide a basis for a better understanding of the molecular mechanism(s) of the secondary channel regulators.

### Mechanistic Implications Resulting From the Differences Between TraR and DksA

DksA, together with ppGpp, activates amino acid biosynthetic pathways and represses rRNA transcription [5],[6]. Prevailing thought presents ppGpp as the mechanistic effector of these aspects of the stringent response because ppGpp levels correlate with the transcriptional effects observed, whereas the levels of DksA remain constant during the growth phases of *E. coli* [5],[6],[44]. DksA is therefore thought to act primarily as a cofactor to stabilize binding of ppGpp to RNAP, enhancing ppGpp effects on transcription initiation [5],[15]. These ideas arose from the ppGpp/RNAP co-crystal structure localizing ppGpp binding near the active center of RNAP in the secondary channel [45]. In addition, in vitro experiments have shown that ppGpp directly affects the *rrnB* P1 rRNA promoter by decreasing the stability and half-lives of RNAP open complexes [46]. We show that TraR can substitute for DksA function in the absence of ppGpp, indicating that ppGpp may not be required, either for positive or negative transcriptional regulation. In addition, recent studies have also suggested that DksA can work independently of ppGpp, fueling the question of exactly how DksA modulates the activity of ppGpp (or vice versa) [7],[8]. Magnusson et al. [7] have shown that high levels of DksA can partially rescue the multiple ppGpp^0^ amino acid auxotrophies observed in a MC4100 background, although these effects were not seen in wild-type MG1655, the strain used in this study. Both in vivo and in vitro, a large excess of DksA over RNAP can repress the *rrnB* P1 promoter in the absence of ppGpp, and in vitro, ppGpp alone has no effect on amino acid biosynthetic promoters [5],[6],[46]. Furthermore, the biological significance of the placement of ppGpp in the original ppGpp/RNAP co-crystal structure is questionable [24]. Based on these findings and our results with TraR, we postulate that DksA may be more than a passive coregulator for at least several promoters during the stringent response.

Since TraR can mimic DksA function in vivo, the ppGpp-independent nature of TraR may reveal several important mechanistic implications for ppGpp and DksA. DksA has two N-terminal α-helices (coiled coil) and is ppGpp-dependent for many processes while TraR may possess only one α-helix and can function independently of ppGpp. Two coils interacting inside the secondary channel would be more spatially restrictive than one. TraR, with only one putative protruding α-helix, would be more dynamic within the secondary channel and more able to adopt a conformation required to modulate transcription.

The structural/functional conservation of the invariant aspartic acid residues of DksA in TraR is of particular interest. When the second conserved aspartic acid residue of TraR was mutated (D6N), the ability of TraR to function both in positively activating amino acid biosynthesis and repressing the *rrnB* P1 promoter was abolished, similar to results seen with DksA on the phage T7 A1 promoter [15]. Surprisingly, the D6N mutation abolished TraR-mediated activation of *livJ* and repression of rRNA transcription in a ppGpp^0^ strain. Since TraR can work without ppGpp, the invariant aspartic acid residues are unlikely to function solely in the coordination of ppGpp and are likely exerting the transcriptional effects in some other manner. Although such speculation remains to be verified, the results presented in this study suggest that only one coil tipped by aspartic residues is sufficient to substitute for DksA function in the absence of ppGpp.

### Possible Roles of TraR in Horizontal DNA Transfer and Pathogenicity

Homologs for *traR* are found in the transfer operons of many naturally occurring transmissible plasmids, including those involved in pathogenicity and multidrug resistance, highlighting selective pressure for an important function(s). To date, neither we or others [29] have identified a role for TraR in F plasmid transfer in *E. coli* (data not shown). This lack of *traR* function might be due to the laboratory domestication of the F factor to constitutively promote conjugation in *E. coli*. TraR may be a broad range host factor important for conjugation in other species or required for transfer in a “wild-type” F (the F plasmid carries a mutation in *finO*, a gene involved in the repression of the transfer operon [47]). In addition, little is known about conjugation in the wild, and it remains to be determined if TraR plays a role in conjugation under more natural conditions. The presence of TraR could also provide indirect fitness advantages for the host during conjugation. Both DksA and ppGpp modulate the cellular responses of autoaggregation and bacterial motility [7],[8]. Both functions can easily be imagined to be important for conjugation (e.g. the quest for a recipient bacteria and maintaining physical contact during DNA transfer). TraR, independently of nutritional stress, may also control these processes. One of the consequences of the stringent response is the reallocation or partitioning of RNAP among promoters in the cell [48],[49]. Here, we show that induction of TraR decreases rRNA synthesis, which encompasses the majority of transcription [33],[34]. This inhibition of rRNA synthesis would free a major portion of RNAP which could be re-assigned, in this case, to the transcription of episomal genes or stress genes induced by the act of conjugation (e.g. periplasmic stress due to pilus formation). Indeed, we have observed a role of TraR in the upregulation of several stress response pathways similar to DksA and ppGpp together (manuscript in preparation) [50]. Finally, genes found in pathogenicity islands are preferentially activated by DksA/ppGpp [12]–[14],[42],[51],[52]. The presence of TraR on congugative plasmids may allow the bacteria to control expression of these genes independently of ppGpp accumulation. Thus, TraR may play an important role in activation of virulence in presence of conjugative plasmids.

TraR may represent a novel and unique member of the growing family of RNAP secondary channel regulators. Its small size compared to DksA and its regulatory differences with respect to ppGpp provide the ability to dissect the functional similarities and differences between the two homologs, providing not only a better mechanistic understanding of TraR and DksA/ppGpp, but that of the other secondary channel regulators as well. Based on the functions of TraR and its presence on conjugative plasmids, we propose that TraR and DksA may have a fitness role during bacteria mating, promoting horizontal gene transfer, and consequently, bacterial evolution. These observations may provide the basis for new studies designed to combat antibiotic resistance and virulence in emerging pathogens.

## Materials and Methods

### Media and Bacterial Growth

Standard methods of *E. coli* genetics were performed [53]. Unless otherwise stated, all work was done at 32°C with either LB medium or M9 medium, supplemented, when required, with sodium citrate (5–20 mM), ampicillin (50 µg/mL), kanamycin (30 µg/mL), chloramphenicol (12.5 µg/mL), tetracycline (3.33 µg/mL with sodium citrate and 10 µg/mL without), glucose (0.1%), casamino-acids (0.3%), and IPTG (0.1 mM). M9 media was always supplemented with FeCl_2_ (10 µM) and thiamine (vitamin B1) (2 µg/mL).

### Bacterial Strains, Plasmids, Mutant Alleles, and Primers

The backgrounds, genotypes, and sources of the strains of *E. coli* and plasmids used in this study are listed in Table S1 (Supplemental Material). Primers for construction of deletion alleles and plasmids are listed in Table S2 (Supplemental Material). Unless otherwise stated, all strains used are derivatives of MG1655. Mutant alleles were moved into this background via standard P1 transduction [53] or linear transformation techniques with subsequent elimination of the drug-resistance marker by FLP recombinase if necessary [54]. Plasmids were constructed and transformed into strains by standard cloning, mutagenesis, and transformation techniques [55]. For *traR* and *dksA*, the Shine-Dalgarno and ORF were amplified from pOX38 and pJK537, respectively. Antibiotic resistance, PCR, DNA sequencing, and/or phenotypic assays were performed for verification of alleles and plasmids.

### Plating Efficiencies of Auxotrophic Strains

Serial dilutions of overnight cultures grown in LB were performed in 10 mM MgSO_4_. Appropriate volumes of the dilutions of interest were then plated on M9-glucose and M9-glucose-casamino-acid plates, both supplemented with IPTG (0.1 mM) and antibiotics when appropriate. The plates were incubated for 4 days at 32°C and colonies were counted. Percentages were obtained from the ratio of colonies growing on M9-glucose vs. the M9-glucose (glu)-casamino-acid (CAA) plates. Errors bars depict 1 standard deviation calculated from 3 independent experiments.

### β-Galactosidase Activity Assays

Overnight cultures were diluted 1/100 and grown aerobically at 32°C in LB supplemented with ampicillin, and, when appropriate, IPTG for plasmid induction. For β-galactosidase assays involving the F plasmid, M9 media supplemented with glucose and tetracycline was used. Samples were taken at appropriate OD_600_ intervals and assayed as previously described [53]. For β-galactosidase activity (per ml), OD_420_×1000 / reaction time vs. OD_600_ was plotted. All graphs are representative of multiple independent experiments that had a maximum variability of 12%. Polynomial (3^rd^ order) regression lines were plotted using Microsoft Excel.

### Motility Assay

Bacterial cells were grown overnight in LB media containing ampicillin, after which each strain was inoculated via toothpick onto low agar (0.375%) LB plates. The plates were incubated at room temperature for ∼24 hours at which the growth halos formed were measured directly [7].

### Measurements of Expression Levels of TraR-His_6_ and DksA-His_6_

Assays were performed as previously described [55]. Briefly, 26 mL cultures of MG1655 [pTraR-His_6_], MG1655 [pDksA-His_6_], and BL21 [pET24-TraR-His_6_] were grown to an OD_600_ of 0.4 at which a 1 mL aliquot was spun down and resuspended in 100 µL of SDS loading buffer. The remaining 25 mL cultures were induced with IPTG (1 mM) for 2 hours after which another 1 mL aliquot was taken, spun down, and resuspended in 100 µL of SDS loading buffer for every OD_600_ 0.4 of culture. Extracts were made of the remaining 24 mL cultures by spinning down and resuspending the pellets in 1 mL of 6 M guanidine, Tris-HCl, (pH 7.4) for every 24 mL of OD_600_ 1.0. TALON 50% slurry resin (100 µL) (Qiagen) was added to 1 mL of the extracts and incubated for 2 hours at 4°C after which the resin was washed twice with the above buffer and once with 6 M Urea, 25 mM Tris-HCl, 500 mM NaCl (pH 7.9) and resuspended in 100 µL of TBS buffer. Samples (15 µL) were then run on 12% polyacrylamide gels and analyzed by Western blotting and Coomassie blue staining. Western blots were performed with antibodies against His_6_ (primary) (1:2000 dilution of His-probe (H-15) rabbit polyclonal IgG, Santa Cruz Biotechnology) and goat anti-rabbit IgG (secondary) (1:2000 dilution of Alex Fluor 647, Invitrogen). The PVDF membrane was scanned with a Typhoon Trio according to the manufacturer (GE).

### Protein Purification

TraR-His_6_ (encoded by pET24-TraR-His_6_ plasmid) was purified with nickel-nitrilotriacetic acid-agarose columns basically as described by Qiagen, except that the binding buffer (BB) was 50 mM NaPO_4_, (pH 8.0), 0.5 M NaCl, 20 mM imidazole, and 10% glycerol. The resin with bound proteins was washed extensively with BB containing 40 mM imidazole, followed by TraR-His_6_ elution with 300 mM imidazole in BB. Pure protein fractions were then dialyzed against storage buffer (10 mM Tris-Cl, (pH 8.0), 0.1 mM EDTA, 0.1 mM DTT, 250 mM NaCl, 50% glycerol). DksA-His_6_ was purified as previously described [22].

### In Vitro Transcription

In vitro transcription reactions were performed as previously described [22]. Briefly, 30 nM RNAP was pre-incubated (25°C) with or without 250 µM ppGpp for 7 min prior to the addition of potassium glutamate (90 mM), and this was followed by a 20 min incubation at 30°C with *rrnB* P1 DNA (10 nM final) and the indicated TraR or DksA concentrations (0–600 nM). The reactions were initiated by adding NTP substrates (100 µM ATP, GTP, and CTP, and 10 µM UTP (10 µCi/reaction [α^32^P]UTP, Amersham Biosciences)) with heparin (100 mg/mL final) and terminated after 8 min by the addition of an equal volume of stop solution (95% formamide, 20 mM EDTA, 0.05% bromophenol blue, and 0.05% xylene cyanol). Samples were analyzed on 7 M urea, 6% polyacrylamide sequencing gels and quantified by phosphorimaging on a GE Healthcare imaging system.

### Supplemental Material

Supplemental material includes: three data figures and legends, a table listing bacterial strains and plasmids used, and a table listing the primers used in this study to construct new deletion alleles and plasmids.

## Supporting Information

Figure S1 TraR, Expressed From the F Plasmid, Inhibits the *rrnB* P1 Promoter. β-galactosidase activity of the *rrnB* P1-*lacZ* promoter fusion performed in M9 glucose media. *traR* (open triangles), as present on the F plasmid, represses *rrnB* P1 activity. Δ*traR* indicated by open circles. Graph is representative of 3 independent experiments. At OD600 0.8, the data had up to 12% variation between experiments and a 1.21±0.01 fold difference in β-galactosidase activity between Δ*traR* and *traR* ^+^ strains. Lower *rrnB* P1-*lacZ* activity reflects the use of minimal media (M9 glucose).
(2.61 MB TIF)

Figure S2 DksA and Control Plasmids, Unlike pTraR, do not Inhibit Growth in Logarithmic Cultures. Figure depicting growth curves (early logarithmic growth) of strains containing pDksA or pControl resulting from successive dilutions in LB containing IPTG (0.1 mM). Cultures with induced pControl or pDksA do not inhibit growth (35, 33, 35 minutes and 36, 36, 37 minutes were the respective doubling times) when treated similarly to a culture with induced pTraR (see Figure 4B).
(2.62 MB TIF)

Figure S3 Simulation of *ctrA401ts*. TraR, Like DksA, Activates Motility and Compensates for ppGpp^0^ Motility Defects. Representative picture of cell motility from cultures inoculated on low agar (0.375%) plates with strains of the indicated genotypes harboring respective plasmids (uninduced). Growth was observed after 24 hours of incubation at room temperature and resulting diameters measured (see Figure 5C).
(5.66 MB TIF)

Table S1 *Escherichia coli* K12 Strains and Plasmids. *Escherichia coli* K12 strains and plasmids used in this study. See references [57]–[62] for the original sources of the plasmids and strains.
(0.07 MB DOC)

Table S2 Primers for Construction of New Deletion Alleles and Plasmids. Primers for construction of new deletion alleles and plasmids used in this study.
(0.02 MB DOC)

## References

[1] Cashel M, Gentry D, Hernandez VJ, Vinella D, Neidhardt FC, Curtiss R, Ingraham EC, Lin ECC, Low KB (1996). The stringent response.. *Escherichia coli* and *Salmonella*: cellular and molecular biology. 2nd ed.

[2] Paul BJ, Ross W, Gaal T, Gourse RL (2004). rRNA transcription in *Escherichia coli*.. Annu Rev Genet.

[3] Magnusson LU, Farewell A, Nystrom T (2005). ppGpp: a global regulator in *Escherichia coli*.. Trends Microbiol.

[4] Brown L, Gentry D, Elliott T, Cashel M (2002). DksA affects ppGpp induction of RpoS at a translational level.. J Bacteriol.

[5] Paul BJ, Barker MM, Ross W, Schneider DA, Webb C (2004). DksA: a critical component of the transcription initiation machinery that potentiates the regulation of rRNA promoters by ppGpp and the initiating NTP.. Cell.

[6] Paul BJ, Berkmen MB, Gourse RL (2005). DksA potentiates direct activation of amino acid promoters by ppGpp.. Proc Natl Acad Sci USA.

[7] Magnusson LU, Gummesson B, Joksimovic P, Farewell A, Nystrom T (2007). Identical, independent, and opposing roles of ppGpp and DksA in *Escherichia coli*.. J Bacteriol.

[8] Aberg A, Shingler V, Balsalobre C (2008). Regulation of the *fimB* promoter: a case of differential regulation by ppGpp and DksA *in vivo*.. Mol Microbiol.

[9] Yamanaka K, Mitani T, Ogura T, Niki H, Hiraga S (1994). Cloning, sequencing, and characterization of multicopy suppressors of a *mukB* mutation in *Escherichia coli*.. Mol Microbiol.

[10] Meddows TR, Savory AP, Grove JI, Moore T, Lloyd RG (2005). RecN protein and transcription factor DksA combine to promote faithful recombinational repair of DNA double-strand breaks.. Mol Microbiol.

[11] Kang PJ, Craig EA (1990). Identification and characterization of a new *Escherichia coli* gene that is a dosage-dependent suppressor of a *dnaK* deletion mutation.. J Bacteriol.

[12] Song M, Kim HJ, Kim EY, Shin M, Lee HC (2004). ppGpp-dependent stationary phase induction of genes on *Salmonella* pathogenicity island 1.. J Biol Chem.

[13] Nakanishi N, Abe H, Ogura Y, Hayashi T, Tashiro K (2006). ppGpp with DksA controls gene expression in the locus of enterocyte effacement (LEE) pathogenicity island of enterohaemorrhagic *Escherichia coli* through activation of two virulence regulatory genes.. Mol Microbiol.

[14] Thompson A, Rolfe MD, Lucchini S, Schwerk P, Hinton JC (2006). The bacterial signal molecule, ppGpp, mediates the environmental regulation of both the invasion and intracellular virulence gene programs of *Salmonella*.. J Biol Chem.

[15] Perederina A, Svetlov V, Vassylyeva MN, Tahirov TH, Yokoyama S (2004). Regulation through the secondary channel-structural framework for ppGpp-DksA synergism during transcription.. Cell.

[16] Orlova M, Newlands J, Das A, Goldfarb A, Borukhov S (1995). Intrinsic transcript cleavage activity of RNA polymerase.. Proc Natl Acad Sci USA.

[17] Erie DA, Hajiseyedjavadi O, Young MC, von Hippel PH (1993). Multiple RNA polymerase conformations and GreA: control of the fidelity of transcription.. Science.

[18] Shaevitz JW, Abbondanzieri EA, Landick R, Block SM (2003). Backtracking by single RNA polymerase molecules observed at near-base-pair resolution.. Nature.

[19] Kettenberger H, Armache KJ, Cramer P (2003). Architecture of the RNA polymerase II-TFIIS complex and implications for mRNA cleavage.. Cell.

[20] Stebbins CE, Borukhov S, Orlova M, Polyakov A, Goldfarb A (1995). Crystal structure of the GreA transcript cleavage factor from *Escherichia coli*.. Nature.

[21] Opalka N, Chlenov M, Chacon P, Rice WJ, Wriggers W (2003). Structure and function of the transcription elongation factor GreB bound to bacterial RNA polymerase.. Cell.

[22] Potrykus K, Vinella D, Murphy H, Szalewska-Palasz A, D'Ari R (2006). Antagonistic regulation of *Escherichia coli* ribosomal RNA *rrnB* P1 promoter activity by GreA and DksA.. J Biol Chem.

[23] Rutherford ST, Lemke JJ, Vrentas CE, Gaal T, Ross W (2007). Effects of DksA, GreA, and GreB on transcription initiation: insights into the mechanisms of factors that bind in the secondary channel of RNA polymerase.. J Mol Biol.

[24] Vrentas CE, Gaal T, Berkmen MB, Rutherford ST, Haugen SP (2008). Still looking for the magic spot: the crystallographically defined binding site for ppGpp on RNA polymerase is unlikely to be responsible for rRNA transcription regulation.. J Mol Biol.

[25] Doran TJ, Loh SM, Firth N, Skurray RA (1994). Molecular analysis of the F plasmid *traVR* region: *traV* encodes a lipoprotein.. J Bacteriol.

[26] Narra HP, Ochman H (2006). Of what use is sex to bacteria?. Curr Biol.

[27] Frost LS, Ippen-Ihler K, Skurray RA (1994). Analysis of the sequence and gene products of the transfer region of the F sex factor.. Microbiol Rev.

[28] Moore D, Wu JH, Kathir P, Hamilton CM, Ippen-Ihler K (1987). Analysis of transfer genes and gene products within the *traB*-*traC* region of the *Escherichia coli* fertility factor, F.. J Bacteriol.

[29] Maneewannakul K, Ippen-Ihler K (1993). Construction and analysis of F plasmid *traR*, *trbJ*, and *trbH* mutants.. J Bacteriol.

[30] Kramer M, Kecskes E, Horvath I (1981). Guanosine polyphosphate production of *Escherichia coli* stringent and relaxed strains in the stationary phase of growth.. Acta Microbiol Acad Sci Hung.

[31] Will WR, Frost LS (2006). Hfq is a regulator of F-plasmid TraJ and TraM synthesis in *Escherichia coli*.. J Bacteriol.

[32] Will WR, Frost LS (2006). Characterization of the opposing roles of H-NS and TraJ in transcriptional regulation of the F-plasmid *tra* operon.. J Bacteriol.

[33] Nomura M, Gourse R, Baughman G (1984). Regulation of the synthesis of ribosomes and ribosomal components.. Annu Rev Biochem.

[34] Bremer H, Dennis P, Neidhardt FC, Curtiss R, Ingraham EC, Lin ECC, Low KB (1996). Modulation of chemical composition and other parameters of the cell by growth rate.. *Escherichia coli* and *Salmonella*: cellular and molecular biology. 2nd ed.

[35] Hernandez VJ, Bremer H (1990). Guanosine tetraphosphate (ppGpp) dependence of the growth rate control of *rrnB* P1 promoter activity in *Escherichia coli*.. J Biol Chem.

[36] Xiao H, Kalman M, Ikehara K, Zemel S, Glaser G (1991). Residual guanosine 3',5'-bispyrophosphate synthetic activity of *relA* null mutants can be eliminated by *spoT* null mutations.. J Biol Chem.

[37] Kundu TK, Kusano S, Ishihama A (1997). Promoter selectivity of *Escherichia coli* RNA polymerase sigmaF holoenzyme involved in transcription of flagellar and chemotaxis genes.. J Bacteriol.

[38] Nickels BE, Hochschild A (2004). Regulation of RNA polymerase through the secondary channel.. Cell.

[39] Adelman K, Yuzenkova J, La Porta A, Zenkin N, Lee J (2004). Molecular mechanism of transcription inhibition by peptide antibiotic Microcin J25.. Mol Cell.

[40] Mukhopadhyay J, Sineva E, Knight J, Levy RM, Ebright RH (2004). Antibacterial peptide microcin J25 inhibits transcription by binding within and obstructing the RNA polymerase secondary channel.. Mol Cell.

[41] Symersky J, Perederina A, Vassylyeva MN, Svetlov V, Artsimovitch I (2006). Regulation through the RNA polymerase secondary channel. Structural and functional variability of the coiled-coil transcription factors.. J Biol Chem.

[42] Lamour V, Hogan BP, Erie DA, Darst SA (2006). Crystal structure of *Thermus aquaticus* Gfh1, a Gre-factor paralog that inhibits rather than stimulates transcript cleavage.. J Mol Biol.

[43] Lamour V, Rutherford ST, Kuznedelov K, Ramagopal UA, Gourse RL (2008). Crystal Structure of *Escherichia coli* Rnk, a New RNA Polymerase-Interacting Protein.. J Mol Biol.

[44] Gralla JD (2005). *Escherichia coli* ribosomal RNA transcription: regulatory roles for ppGpp, NTPs, architectural proteins and a polymerase-binding protein.. Mol Microbiol.

[45] Artsimovitch I, Patlan V, Sekine S, Vassylyeva MN, Hosaka T (2004). Structural basis for transcription regulation by alarmone ppGpp.. Cell.

[46] Barker MM, Gaal T, Josaitis CA, Gourse RL (2001). Mechanism of regulation of transcription initiation by ppGpp. I. Effects of ppGpp on transcription initiation *in vivo* and *in vitro*.. J Mol Biol.

[47] Yoshioka Y, Ohtsubo H, Ohtsubo E (1987). Repressor gene *finO* in plasmids R100 and F: constitutive transfer of plasmid F is caused by insertion of IS*3* into F *finO*.. J Bacteriol.

[48] Barker MM, Gaal T, Gourse RL (2001). Mechanism of regulation of transcription initiation by ppGpp. II. Models for positive control based on properties of RNAP mutants and competition for RNAP.. J Mol Biol.

[49] Jishage M, Kvint K, Shingler V, Nystrom T (2002). Regulation of sigma factor competition by the alarmone ppGpp.. Genes Dev.

[50] Costanzo A, Nicoloff H, Barchinger SE, Banta AB, Gourse RL (2008). ppGpp and DksA likely regulate the activity of the extracytoplasmic stress factor sigmaE in *Escherichia coli* by both direct and indirect mechanisms.. Mol Microbiol.

[51] Haack KR, Robinson CL, Miller KJ, Fowlkes JW, Mellies JL (2003). Interaction of Ler at the *LEE5* (*tir*) operon of enteropathogenic *Escherichia coli*.. Infect Immun.

[52] Olekhnovich IN, Kadner RJ (2007). Role of nucleoid-associated proteins Hha and H-NS in expression of *Salmonella enterica* activators HilD, HilC, and RtsA required for cell invasion.. J Bacteriol.

[53] Miller JH (1992). A short course in bacterial genetics: a laboratory manual and handbook for *Escherichia coli* and related bacteria..

[54] Datsenko KA, Wanner BL (2000). One-step inactivation of chromosomal genes in *Escherichia coli* K-12 using PCR products.. Proc Natl Acad Sci USA.

[55] Sambrook J, Russell DW (2001). Molecular cloning: a laboratory manual..

[56] Jones DT (1999). Protein secondary structure prediction based on position-specific scoring matrices.. J Mol Biol.

[57] Klimke WA, Rypien CD, Klinger B, Kennedy RA, Rodriguez-Maillard JM (2005). The mating pair stabilization protein, TraN, of the F plasmid is an outer-membrane protein with two regions that are important for its function in conjugation.. Microbiology.

[58] Walsh NP, Alba BM, Bose B, Gross CA, Sauer RT (2003). OMP peptide signals initiate the envelope-stress response by activating DegS protease via relief of inhibition mediated by its PDZ domain.. Cell.

[59] Feng GH, Lee DN, Wang D, Chan CL, Landick R (1994). GreA-induced transcript cleavage in transcription complexes containing *Escherichia coli* RNA polymerase is controlled by multiple factors, including nascent transcript location and structure.. J Biol Chem.

[60] Blattner FR, Plunkett G, Bloch CA, Perna NT, Burland V (1997). The complete genome sequence of *Escherichia coli* K-12.. Science.

[61] Casadaban MJ (1976). Transposition and fusion of the *lac* genes to selected promoters in *Escherichia coli* using bacteriophage lambda and Mu.. J Mol Biol.

[62] Yanisch-Perron C, Vieira J, Messing J (1985). Improved M13 phage cloning vectors and host strains: nucleotide sequences of the M13mp18 and pUC19 vectors.. Gene.

