# Supplementary figures and images for: TraR, a Homolog of a RNAP Secondary Channel Interactor, Modulates Transcription
Source: PLoS Genet. 2009 Jan 16;5(1):e1000345. doi: 10.1371/journal.pgen.1000345 (PMC2613031; doi:10.1371/journal.pgen.1000345)

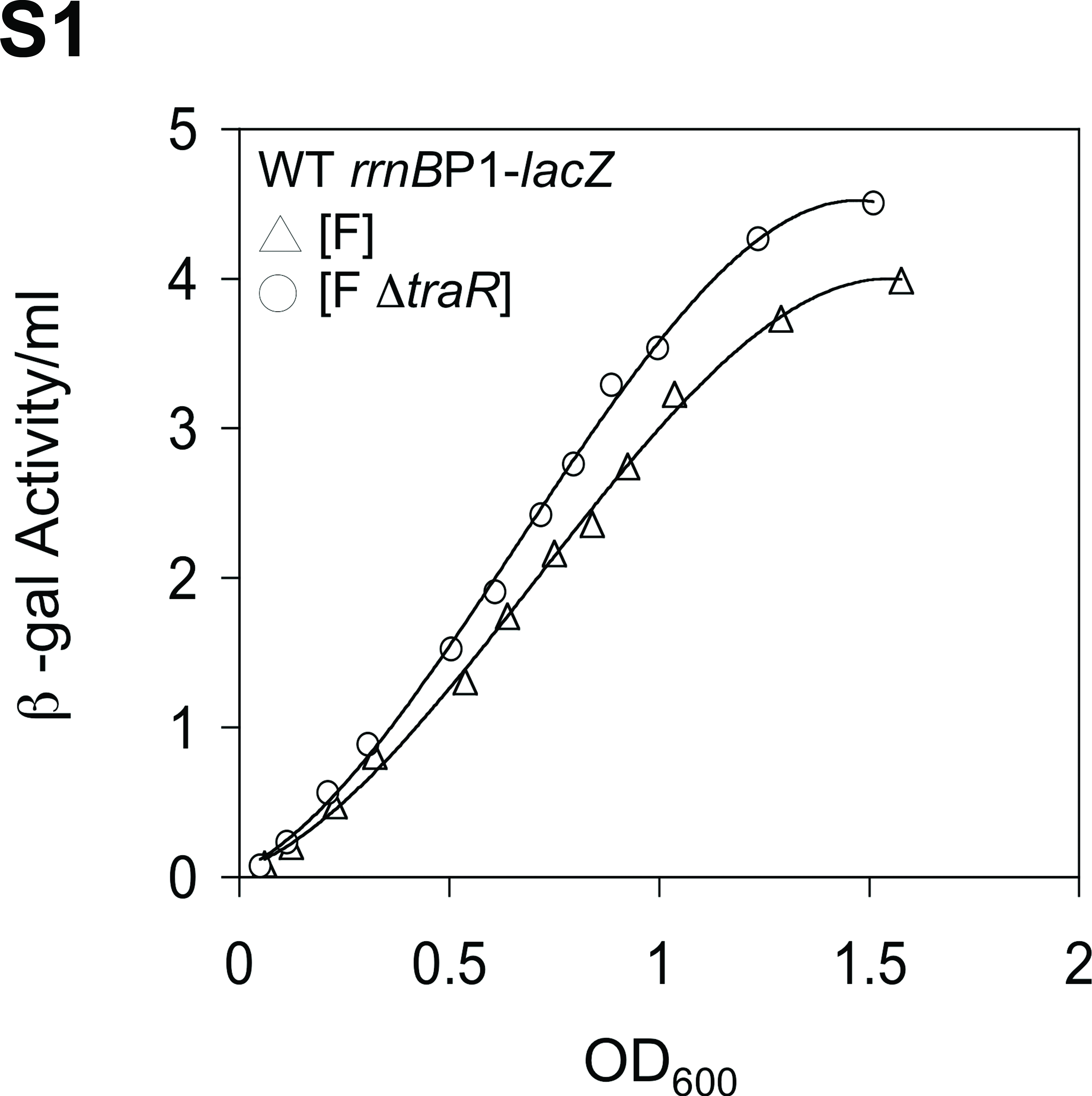

Supplement: Figure S1 — TraR, Expressed From the F Plasmid, Inhibits the rrnB P1 Promoter. β-galactosidase activity of the rrnB P1-lacZ promoter fusion performed in M9 glucose media. traR (open triangles), as present on the F plasmid, represses rrnB P1 activity. ΔtraR indicated by open circles. Graph is representative of 3 independent experiments. At OD600 0.8, the data had up to 12% variation between experiments and a 1.21±0.01 fold difference in β-galactosidase activity between ΔtraR and traR + strains. Lower rrnB P1-lacZ activity reflects the use of minimal media (M9 glucose). (2.61 MB TIF) [file pgen.1000345.s001.tif]

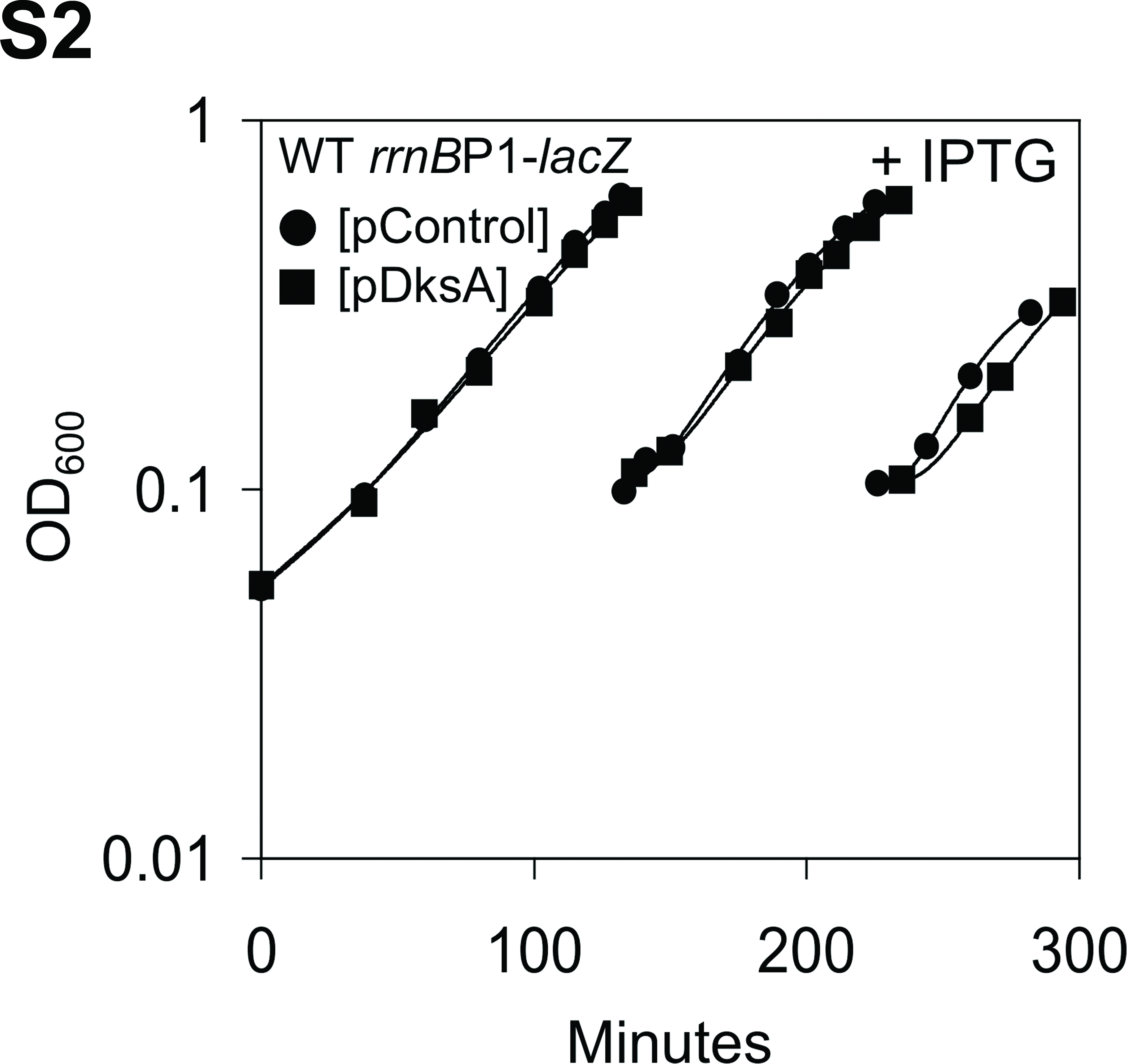

Supplement: Figure S2 — DksA and Control Plasmids, Unlike pTraR, do not Inhibit Growth in Logarithmic Cultures. Figure depicting growth curves (early logarithmic growth) of strains containing pDksA or pControl resulting from successive dilutions in LB containing IPTG (0.1 mM). Cultures with induced pControl or pDksA do not inhibit growth (35, 33, 35 minutes and 36, 36, 37 minutes were the respective doubling times) when treated similarly to a culture with induced pTraR (see Figure 4B). (2.62 MB TIF) [file pgen.1000345.s002.tif]

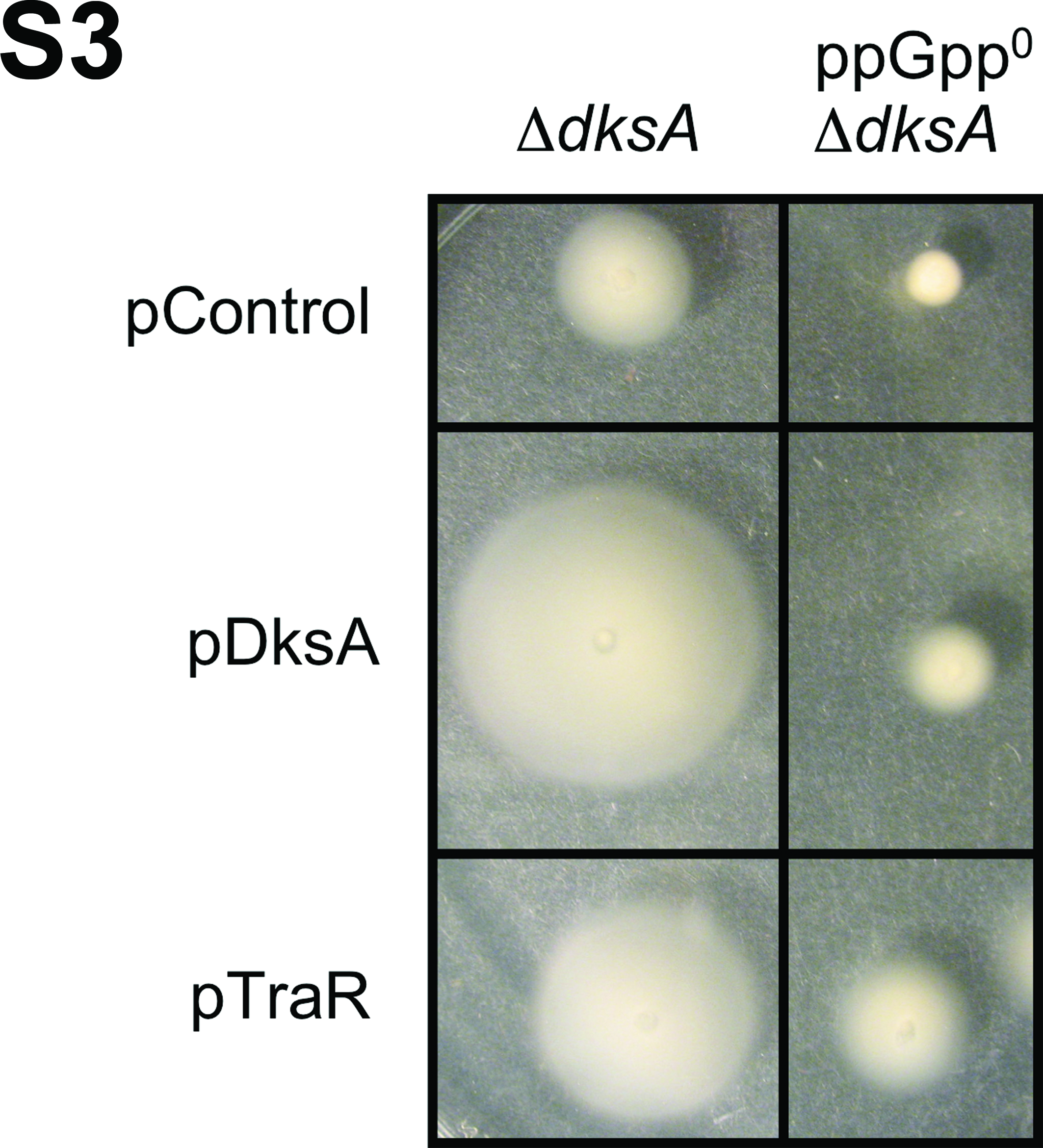

Supplement: Figure S3 — Simulation of ctrA401ts. TraR, Like DksA, Activates Motility and Compensates for ppGpp0 Motility Defects. Representative picture of cell motility from cultures inoculated on low agar (0.375%) plates with strains of the indicated genotypes harboring respective plasmids (uninduced). Growth was observed after 24 hours of incubation at room temperature and resulting diameters measured (see Figure 5C). (5.66 MB TIF) [file pgen.1000345.s003.tif]
