# Supplementary material for: TraR, a Homolog of a RNAP Secondary Channel Interactor, Modulates Transcription
Source: PLoS Genet. 2009 Jan 16;5(1):e1000345. doi: 10.1371/journal.pgen.1000345 (PMC2613031; doi:10.1371/journal.pgen.1000345)
Supplement: Table S1 — Escherichia coli K12 Strains and Plasmids. Escherichia coli K12 strains and plasmids used in this study. See references [57]–[62] for the original sources of the plasmids and strains. (0.07 MB DOC) [file pgen.1000345.s004.doc]

| Table S1. Escherichia coli K12 Strains and Plasmids | | |
| --- | --- | --- |
| Name | Genotype or Relevant Genotype | Reference or Source |
| pCP20 | FLP recombinase vector | [54] |
| pKD3 | Vector used for linear gene replacement | [54] |
| pKD46 | Vector used for linear gene replacement | [54] |
| pOX38 | IncFI, *tra*^+^ *finO^-^*, RepFIA^+^, f1 *Hin*dIII fragment of F | [57] |
| pOX38-Km | Km^R^, pOX38 + *Hin*dIII fragment of Tn*5* | [57] |
| pOX38-Tc | Tc^R^, pOX38::miniTn*10* | [57] |
| pBA169 | pTrc99A Δ*ncoI*, Ap^R^ | [58] |
| pTraR | pBA169/*traR*, Ap^R^ | This study^a^ |
| pDksA | pBA169/*dksA*, Ap^R^ | This study^a^ |
| pTraR-D6N | As pTraR, but mutation of aspartic acid to asparagine (6^th^ residue), Ap^R^ | This study^b^ |
| pBA169-Cm^R^ | pTrc99A Δ*ncoI*, Cm^R^ | This study^c^ |
| pGreB | pGF296, pTrc99C-*greB*, Ap^R^ | [59] |
| pTraR-His_6_ | pBA169/*traR*-*his_6_* (C-terminal), Cm^R^ | This study^a^ |
| pDksA-His_6_ | pBA169/*dksA*-*his_6_* (C-terminal), Cm^R^ | This study^a^ |
| pET24a | His_6_-tagged overexpression vector, Km^R^ | Novagen |
| pET24a-TraR-His_6_ | pET24a/*traR*-*his*_6_ (C-terminal), Km^R^ | This study^a^ |
| pJK537 | *dksA* in pBR322 Ap^R^ | [11] |
| MG1655 | F^-^ λ^-^ *ilvG*^-^ *rfb*-*50 rph*-*1*, sequenced wild-type K12 | [60] |
| MC4100 | F^-^ λ^-^ e*14*^-^ *araD139* Δ(*argF*-*lac*)U*169 rpsL150* *relA1* *f1bB5301 deoC1 ptsF25 rbsR22* Δ(*fruK*-*yeiR*)*725*(*fruA25*) Δ(*fimB*-*fimE*)*632*(::IS*1*) | [61] |
| JM105 | *endA1* *glnV44* *sbcB15 rpsL* *thi*-*1* Δ(*lac*-*proAB*) *hsdR4*(r_K_^-^m_K_^+^) [F' *traD36* *proAB*^+^ *lacI^q^* *lacZ*ΔM15] | [62] |
| BG152 | MG1655 *pyrE*^+^ *lacIZ* λΦ(P*livJ*-lacZ) Δ*relA*::*kan* Δ*spoT*::*cat* | [7] |
| CF6301 | MG1655 *lacIpoZ*Δ(*Mlu*) Δ*relA251*::*kan malB*::*malE*′-*rrnB*P1-φX174*E*′-′*lacZ*-*kan*-′*malK* | [22] |
| CF6306 | MG1655 *lacIpoZ*Δ(*Mlu*) Δ*relA251*::*kan* Δ*spoT207*::*cat* *malB*::*malE*′-*rrnB*P1-φX174*E*′-′*lacZ*-*kan*-′*malK* | Cashel lab |
| CF9240 | MG1655 Δ*dksA*::*tet* | [22] |
| CF12257 | MG1655 *rph*^+^ *lacIpoZ*Δ(*Mlu*) Δ*relA256*::FRT Δ*spoT212*::FRT | Cashel lab |
| CH93 | MG1655 [pKD46] | MG1655 x pKD46 |
| CH256 | MG1655 *lacIpoZ*Δ(*Mlu*) | Lab stock |
| CH918 | MG1655 [pOX38-Km] [pKD46] | CH93 x SMR4067 |
| CH957 | MG1655 *lacIpoZ*Δ(*Mlu*) Δ*dksA*::*tet* | CH256 x P1(CF9240) |
| CH959 | MG1655 *lacIpoZ*Δ(*Mlu*) *malB*::*malE*′-*rrnB*P1-φX174*E*′-′*lacZ*-*kan*-′*malK* | CH256 x P1(CF6301) |
| CH963 | MG1655 [pOX38-Km Δ*traR*::FRT*cat*FRT] | CH918^c^ |
| CH965 | MG1655 *lacIpoZ*Δ(*Mlu*) Δ*dksA*::*tet* [pTraR] | CH957 x pTraR |
| CH968 | MG1655 *lacIpoZ*Δ(*Mlu*) Δ*dksA*::*tet* [pBA169] | CH957 x pBA169 |
| CH980 | MG1655 *lacIpoZ*Δ(*Mlu*) *malB*::*malE*′-*rrnB*P1-φX174*E*′-′*lacZ*-*kan*-′*malK* Δ*dksA*::*tet* | CH959 x P1(CF9240) |
| CH982 | MG1655 [pOX38-Km Δ*traR*::FRT] | CH963 x pCP20 |
| CH985 | MG1655 *lacIpoZ*Δ(*Mlu*) *malB*::*malE*′-*rrnB*P1-φX174*E*′-′*lacZ*-*kan*-′*malK* Δ*dksA*::*tet* [pTraR] | CH980 x pTraR |
| CH987 | MG1655 *lacIpoZ*Δ(*Mlu*) *malB*::*malE*′-*rrnB*P1-φX174*E*′-′*lacZ*-*kan*-′*malK* Δ*dksA*::*tet* [pBA169] | CH980 x pBA169 |
| CH988 | MG1655 *lacIpoZ*Δ(*Mlu*) Δ*dksA*::*tet* [pOX38-Km] | CH957 x SMR4067 |
| CH989 | MG1655 *lacIpoZ*Δ(*Mlu*) Δ*dksA*::*tet* [pOX38-Km Δ*traR*::FRT] | CH957 x CH982 |
| CH1018 | MG1655 [pOX38-Km] | MG1655 x 988 |
| CH1019 | MG1655 [pOX38-Km Δ*traR*::FRT] | MG1655 x 989 |
| CH1037 | MG1655 [pTraR] | MG1655 x pTraR |
| CH1039 | MG1655 [pBA169] | MG1655 x pBA169 |
| CH1074 | MG1655 *rph*^+^ *lacIpoZ*Δ(*Mlu*) Δ*relA256*::FRT Δ*spoT212*::FRT [pTraR] | CF12257 x pTraR |
| CH1076 | MG1655 *rph*^+^ *lacIpoZ*Δ(*Mlu*) Δ*relA256*::FRT Δ*spoT212*::FRT [pBA169] | CF12257 x pBA169 |
| CH1090 | MG1655 *lacIpoZ*Δ(*Mlu*) Δ*dksA*::*tet* [pDksA] | CH957 x pDksA |
| CH1092 | MG1655 *lacIpoZ*Δ(*Mlu*) *malB*::*malE*′-*rrnB*P1-φX174*E*′-′*lacZ*-*kan*-′*malK* Δ*dksA*::*tet* [pDksA] | CH980 x pDksA |
| CH1093 | MG1655 *rph*^+^ *lacIpoZ*Δ(*Mlu*) Δ*relA256*::FRT Δ*spoT212*::FRT [pDksA] | CF12257 x pDksA |
| CH1186 | MG1655 *lacIpoZ*Δ(*Mlu*) *malB*::*malE*′-*rrnB*P1-φX174*E*′-′*lacZ*-*kan*-′*malK* [pTraR] | CH959 x pTraR |
| CH1187 | MG1655 *lacIpoZ*Δ(*Mlu*) *malB*::*malE*′-*rrnB*P1-φX174*E*′-′*lacZ*-*kan*-′*malK* [pDksA] | CH959 x pDksA |
| CH1189 | MG1655 *lacIpoZ*Δ(*Mlu*) *malB*::*malE*′-*rrnB*P1-φX174*E*′-′*lacZ*-*kan*-′*malK* [pBA169] | CH959 x pBA169 |
| CH1213 | MG1655 *lacIpoZ*Δ(*Mlu*) Δ*relA251*::*kan* Δ*spoT207*::*cat* *malB*::*malE*′-*rrnB*P1-φX174*E*′-′*lacZ*-*kan*-′*malK* [pTraR] | CF6306 x pTraR |
| CH1214 | MG1655 *lacIpoZ*Δ(*Mlu*) Δ*relA251*::*kan* Δ*spoT207*::*cat* *malB*::*malE*′-*rrnB*P1-φX174*E*′-′*lacZ*-*kan*-′*malK* [pDksA] | CF6306 x pDksA |
| CH1215 | MG1655 *lacIpoZ*Δ(*Mlu*) Δ*relA251*::*kan* Δ*spoT207*::*cat* *malB*::*malE*′-*rrnB*P1-φX174*E*′-′*lacZ*-*kan*-′*malK* [pBA169] | CF6306 x pBA169 |
| CH1224 | MC4100 [pOX38-Tc] | Via L. Frost |
| CH1269 | MC4100 [pOX38-Tc] [pKD46] | CH1224 x pKD46 |
| CH1275 | MC4100 [pOX38-Tc Δ*traR*::FRT*cat*FRT] | CH1269^c^ |
| CH1288 | MC4100 [pOX38-Tc Δ*traR*::FRT] | CH1275 x pCP20 |
| CH1292 | MG1655 *rph*^+^ *lacIpoZ*Δ(*Mlu*) Δ*relA256*::FRT Δ*spoT212*::FRT Δ*dksA*::*tet* | CF12257 x P1(CF9240) |
| CH1299 | MG1655 *rph*^+^ *lacIpoZ*Δ(*Mlu*) Δ*relA256*::FRT Δ*spoT212*::FRT Δ*dksA*::*tet* [pOX38-Km] | CH1292 x CH1018 |
| CH1300 | MG1655 *rph*^+^ *lacIpoZ*Δ(*Mlu*) Δ*relA256*::FRT Δ*spoT212*::FRT Δ*dksA*::*tet* [pOX38-Km Δ*traR*::FRT*cat*FRT] | CH1292 x CH1019 |
| CH1320 | MG1655 *lacIpoZ*Δ(*Mlu*) *malB*::*malE*′-*rrnB*P1-φX174*E*′-′*lacZ*-*kan*-′*malK* [pOX38-Tc] | CH959 x CH1224 |
| CH1321 | MG1655 *lacIpoZ*Δ(*Mlu*) *malB*::*malE*′-*rrnB*P1-φX174*E*′-′*lacZ*-*kan*-′*malK* [pOX38-Tc Δ*traR*::FRT] | CH959 x CH1288 |
| CH1326 | MG1655 *rph*^+^ *lacIpoZ*Δ(*Mlu*) Δ*relA256*::FRT Δ*spoT212*::FRT Δ*dksA*::*tet* [pTraR] | CH1292 x pTraR |
| CH1327 | MG1655 *rph*^+^ *lacIpoZ*Δ(*Mlu*) Δ*relA256*::FRT Δ*spoT212*::FRT Δ*dksA*::*tet* [pDksA] | CH1292 x pDksA |
| CH1328 | MG1655 *rph*^+^ *lacIpoZ*Δ(*Mlu*) Δ*relA256*::FRT Δ*spoT212*::FRT Δ*dksA*::*tet* [pBA169] | CH1292 x pBA169 |
| CH1511 | MG1655 *pyrE*^+^ *lacIZ* λΦ(P*livJ*-lacZ) [pTraR] | RLG4422 x pTraR |
| CH1512 | MG1655 *pyrE*^+^ *lacIZ* λΦ(P*livJ*-lacZ) [pDksA] | RLG4422 x pDksA |
| CH1513 | MG1655 *pyrE*^+^ *lacIZ* λΦ(P*livJ*-lacZ) [pBA169] | RLG4422 x pBA169 |
| CH1580 | MG1655 *lacIpoZ*Δ(*Mlu*) Δ*relA251*::*kan* Δ*spoT207*::*cat* *malB*::*malE*′-*rrnB*P1-φX174*E*′-′*lacZ*-*kan*-′*malK* [pTraR-D6N] | CF6306 x pTraR-D6N |
| CH1631 | MG1655 *lacIpoZ*Δ(*Mlu*) Δ*dksA*::*tet* [pTraR-D6N] | CH957 x pTraR-D6N |
| CH1904 | MG1655 *pyrE*^+^ *lacIZ* λΦ(P*livJ*-lacZ) Δ*relA*::*kan* Δ*spoT*::*cat* [pDksA] | BG152 x pDksA |
| CH1905 | MG1655 *pyrE*^+^ *lacIZ* λΦ(P*livJ*-lacZ) Δ*relA*::*kan* Δ*spoT*::*cat* [pBA169] | BG152 x pBA169 |
| CH2041 | MG1655 *pyrE*^+^ *lacIZ* λΦ(P*livJ*-lacZ) Δ*dksA*::*tet* | RLG4422 x CF9240 |
| CH2126 | MG1655 *lacIpoZ*Δ(*Mlu*) Δ*dksA*::*tet* [pOX38-Km] [pGreB] | CH988 x pGreB |
| CH2175 | MG1655 *pyrE*^+^ *lacIZ* λΦ(P*livJ*-lacZ) Δ*relA*::*kan* Δ*spoT*::*cat* [pTraR] | BG152 x pTraR |
| CH2176 | MG1655 *pyrE*^+^ *lacIZ* λΦ(P*livJ*-lacZ) Δ*relA*::*kan* Δ*spoT*::*cat* [pTraR-D6N] | BG152 x pTraR-D6N |
| CH2177 | MG1655 *pyrE*^+^ *lacIZ* λΦ(P*livJ*-lacZ) Δ*dksA*::*tet* [pTraR] | CH2041 x pTraR |
| CH2178 | MG1655 *pyrE*^+^ *lacIZ* λΦ(P*livJ*-lacZ) Δ*dksA*::*tet* [pDksA] | CH2041 x pDksA |
| CH2179 | MG1655 *pyrE*^+^ *lacIZ* λΦ(P*livJ*-lacZ) Δ*dksA*::*tet* [pBA169] | CH2041 x pBA169 |
| RLG4422 | MG1655 *pyrE*^+^ *lacIZ* λΦ(P*livJ*-lacZ) | Via R. L. Gourse |
| SMR4067 | MC4100 [pOX38-Km] | L. Frost via SM Rosenberg |
| ^a^ Plasmids constructed *via* PCR and standard cloning using primers in Table S2.  ^b^ Plasmid constructed *via* site-directed mutagenesis using primers in Table S2.  ^c^ PCR products, from primers in Table S2, were recombined into the indicated strains. | | |
