# Supplementary material for: TraR, a Homolog of a RNAP Secondary Channel Interactor, Modulates Transcription
Source: PLoS Genet. 2009 Jan 16;5(1):e1000345. doi: 10.1371/journal.pgen.1000345 (PMC2613031; doi:10.1371/journal.pgen.1000345)
Supplement: Table S2 — Primers for Construction of New Deletion Alleles and Plasmids. Primers for construction of new deletion alleles and plasmids used in this study. (0.02 MB DOC) [file pgen.1000345.s005.doc]

| Table S2. Primers for Construction of New Deletion Alleles and Plasmids | |
| --- | --- |
| New Allele or Plasmid | Primers Used |
| Δ*traR*::  FRT*cat*FRT | 5'-GGTAGCGTGTCAGAACATGTCGGGAGGAATATTCCGTGAGTGATGAAGCCGT  GTAGGCTGGAGCTGCTTCG-3'  5'-CATCCGGAATTAAATCACTATGTTCTGCGACTGACTTATGCATAATGTTTCAT  ATGAATATCCTCCTTA-3' |
| pBA169-Cm^R^ | 5'-CTAAATACATTCAAATATGTATCCGCTCATGAGACAATAACCCTGCATATGA  ATATCCTCCTTAG-3'  5'-GAGTAAACTTGGTCTGACAGTTACCAATGCTTAATCAGTGAGGCGTGTAGGC  TGGAGCTGCTTC-3' |
| pTraR | 5'-GCCGAATTCGTGTCAGAACATGTCGGG-3'  5'-GGCAAGCTTTCACTATGTTCTGCGAC-3' |
| pDksA | 5'-GCCGAATTCCGATAGTGCGTGTTAAGG-3'  5'-GGCAAGCTTGGTAAACGTGATGGAACGG-3' |
| pTraR-D6N | 5'-CCGTGAGTGATGAAGCCAATGAAGCATATTCAGTGAC-3'  5'-GTCACTGAATATGCTTCATTGGCTTCATCACTCACGG-3' |
| pET24-TraR-His_6_ | 5'-GGCAAGCTTTTAGTGATGGTGATGGTGATGTGCATAATGTTTTCTCTGTCTTTCC-3'  5'-GCCGAATTCATTAAAGAGGAGAAATTAACTATGAGTGATGAAGCCGATGAAGC-3' |
| pTraR-His_6_ | 5'-GCCGAATTCGTGTCAGAACATGTCGGG-3'  5'-GGCAAGCTTTTAGTGATGGTGATGGTGATGTGCATAATGTTTTCTCTGTCTTTCC-3' |
| pDksA-His_6_ | 5'-GCCGAATTCCGATAGTGCGTGTTAAGG-3'  5'-GGCAAGCTTTTAGTGATGGTGATGGTGATGGCCAGCCATCTGTTTTTCGCG-3' |
